# The First Virome of a Russian Vineyard

**DOI:** 10.3390/plants12183292

**Published:** 2023-09-18

**Authors:** Svetlana Vinogradova, Elena Porotikova, Emiliya Navrotskaya, Zsuzsanna Nagyne Galbacs, Sébastien Massart, Eva Varallyay

**Affiliations:** 1 Institute of Bioengineering, Research Center of Biotechnology of the Russian Academy of Sciences, Leninsky Prospect 33, 119071 Moscow, Russia; plantvirus@mail.ru (E.P.); emiliya.navrotskaya@mail.ru (E.N.); 2 Genomics Research Group, Department of Plant Pathology, Institute of Plant Protection, Hungarian University of Agriculture and Life Sciences, Szent-Gyorgyi Albert Street 4, H-2100 Godollo, Hungary; nagyne.galbacs.zsuzsanna@uni-mate.hu (Z.N.G.); varallyay.eva@uni-mate.hu (E.V.); 3 Laboratory of Integrated and Urban Phytopathology, TERRA, Gembloux Agro-Bio Tech, Liège University, 5030 Gembloux, Belgium; sebastien.massart@uliege.be

**Keywords:** *Vitis vinifera*, grapevine viruses, viral metagenomics, high-throughput sequencing, virome, mycoviruses

## Abstract

Among other pathogens, more than 80 viruses infect grapevine. The aim of this work was to study the virome diversity of grapevine viruses and mycoviruses of a vineyard using high-throughput sequencing technologies. The grapevine virome was studied in symptomatic vines of the Rkatsiteli cultivar (*V. vinifera*) collected at the vineyards of the Krasnodar Krai in Russia. Ribosomal-depleted total RNA and isolated small RNAs were used for library preparation and high-throughput sequencing. Six grapevine-infecting viruses and two viroids were validated by RT-PCR and analyzed phylogenetically. We identified the presence of grapevine leafroll-associated virus 3, grapevine Pinot gris virus, grapevine virus T, grapevine rupestris stem-pitting-associated virus, grapevine fleck virus, and grapevine rupestris vein feathering virus, as well as two viroids, grapevine yellow speckle viroid 1 and hop stunt viroid. We also studied the mycovirome of the vineyard and identified nine viruses with single-stranded positive-sense RNA genomes: alternaria arborescens mitovirus 1, botrytis cinerea mitovirus 1, botrytis cinerea mitovirus 2, botrytis cinerea mitovirus 3, botrytis cinerea mitovirus 4, sclerotinia sclerotiorum mitovirus 3, botrytis cinerea hypovirus 1, grapevine-associated narnavirus 1, and botrytis virus F. In addition, we identified botrytis cinerea hypovirus 1 satellite-like RNA and two single-stranded negative-sense RNA viruses. This is the first study of grapevine mycoviruses in Russia. The obtained result will contribute to the development of biocontrol strategies in the future.

## 1. Introduction

Plants, like all living organisms, are associated with a large number of microorganisms throughout their entire life cycle. They correspond to both endophytic and epiphytic microorganisms, including pathogenic bacteria, phytoplasmas, fungi, oomycetes, and viruses. Plant viral diseases are infectious diseases that lead to significant economic losses in agriculture and require constant monitoring to prevent their spread to new territories [1,2]. Improving detection methods helps to minimize the risks of spreading viruses with planting materials [1].

The development of molecular biology tests and rapid advances in sequencing technologies, in particular high-throughput sequencing (HTS) technologies, have led to the availability of powerful new tools for identifying plant pathogens and studying the microbial community (including viruses) of investigated plants. Protocols based on HTS technologies can decipher the composition of microbial populations in any studied sample. The application of HTS technologies is particularly advanced for plant-virus detection and is currently envisioned as an additional test to evaluate the phytosanitary status of plant material in the frame of certification or quarantine analyses [3].

Grapevine is one of the most important crops in the world; it is affected by more than 80 known viral pathogens. Since the beginning of the use of HTS in the field of grapevine virology, several new viruses, such as grapevine red-blotch-associated virus (GRBaV), grapevine Pinot gris virus (GPGV), and grapevine Syrah virus 1 (GSyV-1), have been discovered, and the pathogenicity of some of them have been demonstrated [4,5,6].

In addition to the detection of grapevine viruses, HTS makes it possible to survey its virome as a microecosystem comprising viruses of other organisms living on plants, such as fungi and oomycetes. The long co-evolution of pathogenic fungi and oomycetes of grape and their viruses made it possible to established both symbiotic and antagonistic relationships between them. Viral infection in the fungal hosts could be associated with a weakening of the virulence of the fungi, a reduction in the epidemics due to a decrease in the number of spores, and a slowdown in the growth rate of mycelium [7].

The HTS of plant tissue is an important tool in the study of grapevine-associated fungal and oomycete viruses, as these mycoviruses are obligate parasites and often challenging to culture. As early as 2011, the grapevine virome has been shown to be dominated by mycoviruses [8]. In a number of studies of grapevine fungi using the HTS of total and small RNAs, the phytosanitary status of grapevine plants was examined, and new mycoviruses were discovered, for instance, botrytis ourmia-like virus (BOLV, ss(+) RNA, *Botourmiaviridae*) and botrytis cinerea negative-stranded RNA virus 1 (BcNSRV-1, ss(−) RNA unclassified ssRNA negative-strand viruses) that infect the phytopathogenic fungus *Botrytis* [9,10,11,12]. Another study of grapevine *Botrytis* identified 92 mycoviruses, including 62 putatively new species [13]. The virome analysis of culturable wood fungal endophytes in esca-symptomatic and -asymptomatic grapevines identified 38 putative new viral species [14]. Another study of grapevine samples infected with *Plasmopara viticola* allowed for the identification of 283 new RNA viruses [15]. Although, over the past few years, metagenomics studies have made it possible to identify a large number of new viruses, the determination of their taxonomic position by ICTV is still in progress.

In our previous study of Russian commercial vineyards, we carried out large-scale phytosanitary monitoring in three vine growing regions. It resulted in the detection of seven economically significant grapevine viruses by RT-PCR in 54.5% of the 1857 investigated samples [16]. In a follow-up study, we identified an additional six viruses and 4 viroids using small RNA (sRNA) HTS upon discribing the viromes of 38 vines [17].

In this study, we characterized the virome of a vineyard in Russia using HTS technologies on total and small RNAs. We identified six grapevine viruses and two viroids: grapevine leafroll-associated virus 3, grapevine Pinot gris virus, grapevine virus T, grapevine rupestris stem-pitting-associated virus, grapevine fleck virus, grapevine rupestris vein feathering virus, grapevine yellow speckle viroid 1, and hop stunt viroid. In addition, we detected infections of 11 grapevine-associated mycoviruses.

## 2. Results

### 2.1. Symptoms in the Vineyard

During the phytosanitary monitoring of a vineyard in the Krasnodar Krai in Russia of the Rkatsiteli cultivar, plants with partial or complete decline were observed (Supplementary Table S1). They were characterized by a decrease in the size of the leaves or their complete disappearance, shortened shoots with short internodes, and zigzag growth. In addition, symptoms of leafroll and deformations of the leaf blade, as well as the appearance of yellow dots and circles along the veins and over the entire surface of the leaves were observed. On bunches in the BBCH 79 phenophase, an uneven size of berries were observed. Eight individuals showing the above symptoms were selected and pooled for HTS analysis.

### 2.2. Bioinformatic Analysis of HTS

#### 2.2.1. Ribosomal-Depleted RNAseq

As a result of the sequencing of the ribosomal-depleted RNA library (rRNA-depleted RNAseq), 10.6 million raw paired-end reads were obtained (Supplementary Table S2). After trimming and quality control, about 3 million high-quality nonredundant reads were retained. Among them, 1 million reads remained after grapevine genome substraction. These reads were assembled using SPAdes into 20,884 (N50 = 1526 bp) and 9243 contigs (N50 = 950 bp), with and without grapevine reads, respectively.

The number of viruses and viroids identified by annotating the contigs in the analysis of total RNA HTS data with and without grapevine reads was the same for both SPAdes and Geneious assemblers (Table 1, Supplementary Table S3). All viruses were consistently identified by both assemblers and by blastn and tblastx, in contrast to viroids, which were identified only with the assembly by Geneious. In general, the percentage of reference genome coverage varied from 78.2% for GFkV to 100% for GLRaV-3 and viroids. The complete genome was obtained *de novo* only for GLRaV-3. Therefore, iterative mapping of the reads to the nearest or reference genome and extending the obtained contigs helped to assemble complete genomes for GPGV, GVT, GRSPaV, and GRVFV.

As we sequenced total RNA of eight samples in one pool and then validated all viruses except GVT in more than one sample, we were able to obtain a chimeric genome from several virus isolates upon assembly. To test this hypothesis, trimmed reads were mapped to the reference genome from Genebank with custom sensitivity and with 5% mismatches per read compared to the 20% with medium-low mapping sensitivity (Supplementary Table S4). The number of ambiguous nucleotides in GLRaV-3 genome assembled from the sequenced pool sample correspond to three bases (0.016% of the consensus sequence length) with both mapping parameters. The pairwise alignment of genomes consensus sequences obtained as result of mapping with different parametes show their identity. For GPGV, the number of ambiguous genomes with medium-low and custom sensitivity were 14 (0.193%) and 11 (0.152%), respectively. Consensus sequences differed by one nucleotide. For GYSVd, no reads were mapped to the reference sequence with custom sensetivity, so we compared different parameters by mapping the trimmed reads to the closest isolate from GenBank. We found two (0.546%) ambiguities in the consensus sequence with both parameters, and these consensus sequences were identical. In the HSVd genome, we did not observe any ambiguities; the consensus sequences were identical. Based on this, we assume the presence of one isolate in the pool for each of the viruses and viroids: GLRaV-3, GPGV, GYSVd-1, and HSVd/. In addition, GVT was validated in one sample, so its complete genome is a single isolate.

A significantly smaller number of reads was mapped to the reference GRSPaV, GFkV, and GRVFV genomes at maximum mismatch of 5%. Taking into account the number of ambiguities (4.2–7.5%), the existence of several isolates in the pool is possible. Therefore, we did not analysed these genomes during recombination and phylogenetic analysis.

#### 2.2.2. sRNAseq

As a result of the small RNA sequencing (sRNAseq), 15.8 million raw reads were obtained (Supplementary Table S2). After preprocessing, about 2 million reads remained in the analysis with grapevine reads, and about 1 million remained in the analysis without grapevine reads. Using Geneious for assembly, 170,431 contigs (but N50 = 57 bp) and 96,586 contigs (N50 = 55 bp) were obtained using the reads with and without grapevine-specific reads, respectively. The number of contigs assembled using Velvet was lower. In total, 3825 (N50 = 45 bp) and 989 (N50 = 49 bp) contigs with and without grapevine reads were obtained using Velvet. 

As a result of the blastn analysis of contigs assembled by Geneious with grapevine reads, all viruses and viroids, except GVT, were detected, whereas without grapevine reads, GVT, GFkV, and GRVFV were not detected (Supplementary Table S3), suggesting the unspecific depletion of viral reads during the genome subtraction step. When mapping trimmed reads to the reference genomes of viruses identified by contig BLAST, we obtained high coverage of the genome for all viruses and viroids, except GVT, with and without grapevine reads. 

### 2.3. Validation and Phylogenetic Analysis of Grapevine Viruses

The presence of all viruses and viroids detected during the bioinformatic analysis of HTS data was validated by RT-PCR with each individual plant used in the sample pool for HTS analysis (Table 1).

GLRaV-3 (family Closteroviridae, genus *Ampelovirus*) was identified in two samples. In the dendrogram, its complete genome with high bootstrap support clustering with samples from USA, Brazil, South Africa, and Canada. According to its clustering with representative isolates in the dendrogram constructed for the coat protein genes, cut from the complete genome sequence, we assigned the identified isolate to genetic group I (Supplementary Figures S1 and S2). The identity with the closest isolate (MH814489.1) from the GenBank was 99.7%.

The most prevalent virus in the analyzed samples was GPGV (family Betaflexiviridae, genus *Trichovirus*), which was identified in eight samples. In the dendrogram, GPGV clustered with isolates from France (Supplementary Figure S3). The BLASTn analysis showed that its identity with the nearest isolate (MK514528.1) from the GenBank was 99.2%.

To validate GVT (family Betaflexiviridae, genus *Foveavirus*) detection, we designed primers GVT_F1 and GVT_R based on the sequence of our contigs to amplify a partial sequence of the coat protein. The presence of GVT was confirmed in one sample (Supplementary Table S5). As a result of the phylogenetic analysis of the complete genomic sequences of the virus, the identified isolate was shown to cluster with the 99% bootstrap support next to the isolates from Italy and France and to belong to molecular group IV out of seven groups described previously (Supplementary Figure S4) [18]. The BLASTn analysis showed that its identity with the nearest isolate from the GenBank (MZ440736.1) was 96.7%.

GRSPaV (family Betaflexiviridae, genus *Foveavirus*), GFkV (family Tymovidae, genus *Maculavirus*), and GRVFV (family Tymovidae, genus *Marafivirus*) were detected in seven, five, and three samples, respectively. As complete genomes were not obtained for these viruses, we did not conduct phylogenetic analysis.

The GYSVd-1 viroid was validated in two samples, and its genome clustered with Type 1 genomes on the phylogenetic tree (Supplementary Figure S5). HSVd was detected in all samples and clustered most closely with the isolates of the Hop group (Supplementary Figure S6).

As a result of the analysis, recombination points were not found in the complete genomes of the Russian isolates GLRaV-3, GVT, GPGV, HSVd, and GYSVd-1.

### 2.4. Mycovirus Analysis

Contigs assembled from the rRNA depleted RNAseq using SPAdes from all trimmed reads, including grapevine specific reads, were analyzed using blastn and tblastx against a reference database of viral nucleotide sequences. As a result of blastn, we identified contigs of 13 mycoviruses from five families (Table 2). The Tblastx analysis of the same contigs made it possible to identify the contigs of 22 mycoviruses from 10 families. Among them, there were 15 ss(+) RNA virus species, three ss(−) RNA, two dsRNA, one ssRNA reverse-transcribing viruses, and one species corresponding to RNA satellites (Supplementary Table S6).

Among the 15 ss(+) RNA species, nine belonged to the genus *Mitovirus*. For alternaria arborescens mitovirus 1 (AaMV-1), botrytis cinerea mitovirus 1, botrytis cinerea mitovirus 2, botrytis cinerea mitovirus 3, botrytis cinerea mitovirus 4, and sclerotinia sclerotiorum mitovirus 3, the coverage of the reference sequence was 96.5–99.1%. All of these viruses were validated by RT-PCR. The most common was AaMV-1; it was identified in five samples. We also validated two more ss(+) RNA species: grapevine associated narnavirus-1 (its complete genome was also assembled) and botrytis virus F. They were identified in three and two grapevine samples, respectively. Botrytis cinerea hypovirus 1 satellite-like RNA was identified in two samples.

As a result of tblastx, we identified contigs similar to three species of ss(−) RNA. However, when the library reads were mapped to the reference sequences of these mycoviruses, we did not identify any reads. An analysis using the NCBI BLASTn tool showed the identity of these contigs with several other ss(−) RNA viruses. The mapping of library reads to these sequences showed that the genome coverage of two of them, *Botrytis cinerea negative-stranded RNA virus 3* and *Sclerotinia sclerotiorum negative-stranded RNA virus 9*, corresponded to 96.2 and 98.6%, respectively. These viruses were not identified by tblastx as their reference sequences were absent in the refseq database of reference viral genomes. We designed primers for the identified contigs and sequenced the amplification products. The resulting amplicons were mapped to the reference sequences of both of these viruses. These viruses have similar parts of their genomes and have not yet been classified by ICTV. To determine the taxonomic affiliation of the viruses we have identified, a more detailed study of their reference sequences is required.

In addition to viruses whose presence were identified and validated using molecular biology methods, we found contigs similar to several ss(+) RNA and dsRNA viruses (Supplementary material “Analysis of mycoviruses contigs by NCBI Blastn tool”). When the library reads were mapped to the nucleotide sequences of the nearest isolates, they were found to have low coverage. This indicates that these reads belong to other closely related viruses or to species not yet described, for which we did not obtain a sufficient number of reads to assemble a complete sequence. For six of them (mycoviruses of *P. viticola* and *Erysiphe necator*), we confirmed their presence by RT-PCR and Sanger sequencing to obtain partial genome sequences (see Supplementary Table S7 and Supplementary material “Validation of additional mycoviruses”). 

Thus, we identified viruses of the main grapevine-associated fungi: *Alternaria* sp., one virus species; *Botrytis cinerea* (causing gray mold disease), eight species; *Sclerotinia sclerotiorum* (white mold), two species; as well as contigs of *P. viticola* (downy mildew) and *E. necator* (powdery mildew) viruses (Table 2).

## 3. Discussion

The aim of this study was to determine and analyze the virome of a vineyard using a metagenomics approach to obtain the most complete information on both plant and fungal viruses. For the first time in Russia, we identified several mycoviruses.

To determine the most suitable approach for grapevine virome analysis for our samples, we used the HTS of plant starting from total-ribosomal-depleted RNA and small RNAs. When comparing our results of virome analysis using HTS of total RNA and small RNAs, from the point of view of the most complete description of the virome, the analysis of total RNA is more appropriate in terms of the number of pathogens detected and the ability to assemble complete genomes. Based on the raw data we obtained, we were able to assemble complete genomes only using the HTS of total RNA, nevertheless, in other studies, the reconstruction of complete genomes using HTS of sRNA was possible [6,19,20]. In our case, sequencing more reads might be needed for genome reconstruction by sRNA. Some viruses, including GVT, due to their biology or the suppression of RNA-silencing in the plant, are consistently detected only by total RNA, which is confirmed by previous studies [21,22,23,24].

For the analysis of our data on known viral species, the pipeline with the assembly of preprocessed reads (both with and without grapevine reads) by the Geneious assembler and subsequent blastn analysis was optimal. This was the only pipeline that identified most viruses and viroids. However, depending on the aims of the study, for example, when studying diseases of unknown etiology, the best pipeline would be sequencing the sample with RNAseq, then the assembly of the reads using SPAdes and Geneious assemblers, followed by blastn and tblastx analysis. In addition, parameters and thresholds for tools and algorithms used for bioinformatic analysis as well as a reference database are of particular importance [25,26].

Mapping the trimmed reads to known viral genomes of the studied host plant was also effective for detection since all viruses were detected in the analysis of data with grapevine reads, and almost all were detected in the analysis of small RNAs and total RNA without host plant reads. The least efficient for our small RNA data was the pipeline with the elimination of grapevine reads and the tblastx analysis of contigs by any of the assemblers we used. Thus grapevine genome elimination is not an efficient step of the pipeline for our small RNA data analysis. 

As a result of the analysis of the rRNA-depleted RNAseq library, we obtained the complete genomes of GLRaV-3, GPGV, GVT, which made it possible to study their phylogenetic relationships with other sequenced isolates. For several viruses, we observed the same molecular groups as in our previous study using the coat protein gene. For example, the presence of the GLRaV-3 isolates of molecular group I that we previously found in the Republic of Crimea and Krasnodar Krai was confirmed [16,17].

The HTS of several samples in a pool has advantages as it reduces the cost of virome analysis. However, this approach complexifies the reconstruction of viruses genomes and casts doubt on obtaining of complete genomes for each isolate without a detailed bioinformatic analysis of the data. In our pooled samples, we showed the presence of one isolate of GLRaV-3 and GPGV. However, for GRSPaV and GRVFV, the resulting complete genomes could be chimeric. At the same time, it is worth mentioning that generating a chimeric genome is also a risk when sequencing individual samples as several isolates can infect a single plant. For example, according to our previous data from the study of 65 grapevine viromes with GRSPaV, GRVFV, and GFkV, complete genomes of these viruses usually are not assembled *de novo* into one contig [27,28]. The reason for this seems to be the high genetic diversity of the viruses in the individual plant.

In this study of the rRNA-depleted RNAseq library, we identified mycoviruses of the most common necrotrophic pathogenic fungi infecting grapes: *Alternaria* sp., *Botrytis* sp., *Sclerotinia* sp., as well as contigs of viruses of the biotrophic oomycete *P. viticola* and the fungus *E. necator*. Given the large amount of recent data from metagenomic studies, the resulting systematic complexity, and the difficulties in identifying biotrophic viruses, it is challenging to determine the exact mycovirus composition in the plant samples. We identified and confirmed the presence 11 viruses, as well as detected the presence of several contigs that appear to be related to biotrophic host pathogens. Our study clearly shows the advantages of the metagenomics approach that we used, in which we omit the stage of isolation of fungi into pure culture and can identify both mycoviruses and plant viruses in the same experiment and use this first look at the vineyard for a further, more detailed study of the identified fungi and their viruses.

The vines of the characterized vineyard clearly showed symptoms of viral infection. For the study, individuals showing different, but strong symptoms have been chosen to increase the possibility of virus detection. Using two different types of HTS for the virome determination of the pooled sample combined with the validation of every single individual using primers, which are able to detect the identified strains, allow us to characterize not only the virome of the vineyard, but also virome of the sampled vines. In some cases, our results show the presence of a less-viral agent in the sampled plant than we initially expected. HTS is very sensitive, being to detect all viral pathogens in the sample. However, HTS was performed on the pooled sample, and the virome of the individuals were determined by RT-PCR based on the HTS. To avoid a failure in the validation before RT-PCR, we checked if all of our primers could anneal to the identified strain of the particular virus. In some cases in contrast to the presence of obvious symptom we could not detect the suspected pathogen. We assume that our combined diagnostic methods were sensitive enough to detect even traces of the viruses, and it is very unlikely that we failed to detect any presenting pathogens. The causative agent of the symptom in these cases could be a combination of several different viral and fungal pathogens, which could be further altered by abiotic factors. Our research highlights the still-open question: does the missing link of biology and the contribution of HTS-described viruses detail the impact of coinfections on grapevine health and their role in diseases, which would be important to further investigate in the future? 

## 4. Materials and Methods

### 4.1. Plant Samples and Library Construction

The monitoring of white berry Rkatsiteli cultivar (*V. vinifera*) (Georgian selection) in a vineyard located in Krasnodar Krai in Russia was conducted in July 2018. The age of the vineyard was about 20 years. In regard to planting the vineyard, locally produced planting material was used. Leaf and vein samples were collected from 8 symptomatic plants and used for total RNA extraction according to the protocol given Morante-Carriel et al. (2014) [29]. A washing step for highly hydrated tissues was excluded from the protocol. The quality of RNA was checked using an Eppendorf BioSpectrometer Basic and electrophoresis on 1% agarose gel. These samples were used for the construction of total RNA and small RNA libraries.

For total RNA library construction, RNA samples were digested with DNAse I (Thermo Fisher Scientific, Waltham, MA, USA) according to the manufacturer’s protocol, followed by phenol–chloroform extraction and ethanol precipitation. One μg of each sample was pooled and used for library preparation. Ribosomal RNA was removed using a RiboMinus Plant Kit for RNA-Seq (ThermoFisher Scientific, Waltham, MA, USA). Ribosomal-depleted RNA libraries were then prepared using a simplified protocol (no enrichment in poly-A RNA) using a TrueSeq Stranded mRNA kit (Illumina, San Diego, CA, USA). More precisely, ribosomal-depleted RNA entered the process at point 12 of the manufacturer’s instructions; a “Make RFP” chapter, and only Fragment, Prime, Finish mix was added. All further steps were performed according to the instructions [30]. Library quality control was carried out using a Bioanalyzer 2100 (Agilent Technologies, Santa Clara, CA, USA). Paired-end sequencing (2 × 150 bp) was carried out using a Nextseq 500 (Illumina, San Diego, CA, USA).

For small RNA extraction, 5 μg of each total RNA sample was pooled and used for sRNA purification and library preparation using the TruSeq Small RNA Library Preparation Kit (Illumina) according to the manufacturer’s protocol with the modifications described by Czotter et al. (2018) [31]. Library quality control was carried out using a Bioanalyzer 2100 (Agilent Technologies, Santa Clara, USA). The sequencing of single-end (50 bp) was carried out using a HiScanSQ by UD-Genomed (Debrecen, Hungary). Fastq files of the sequenced libraries were deposited in the SRA and can be accessed with the accession numbers RJNA786241 (for rRNA-depleted RNAseq) and RJNA786077 (for sRNAseq).

### 4.2. Bioinformatics Analysis of HTS 

#### 4.2.1. rRNA-Depleted RNAseq

The total RNAseq library was analyzed using Geneious Prime v. 2021.2.2 (Biomatters, Auckland, New Zealand) (Geneious Bioinformatics Software for Sequence Data Analysis. Available online: https://www.geneious.com/ (accessed on 10 May 2022) [32]. As a first step, reads were trimmed using a BBDuk Trimmer, and duplicated reads were removed. The remaining reads were paired and merged. Then, merged and unmerged paired reads were assembled into contigs using SPAdes (with default parameters) and Geneious (medium-low sensitivity parameter for assembly). In parallel, host reads were eliminated from preprocessed reads by mapping them to the grapevine reference genome (GCF_000003745). Unmapped reads were assembled using SPAdes and Geneious. Then, we performed blastn and tblastx searches against the NCBI database of reference viral genomes (refseq release date of 5 May 2022). Subsequently, we selected contigs corresponding to plant or fungi viruses and viroids with an E-Value cutoff of <e^−40^. For the mycovirus identification, we used only the blastn and tblastx analysis of contigs assembled using SPAdes without the elimination of grapevine reads. To determine the number of reads of detected viruses and viroids and the percentage of the reference genome coverage, the preprocessed reads were mapped to reference genomes using the Genious mapper with a medium-low sensitivity parameter and 5 iterations. We also determined the percentage of identity with the closest genome using the NCBI blastn tool. The assembled genomes were deposited in the GenBank (www.ncbi.nlm.nih.gov/genbank/ (accessed on 10 May 2022) (Supplementary Table S8) [33].

#### 4.2.2. sRNAseq

To analyze the small RNA library, we used the Geneious Prime v. 2020.0.4. Reads were trimmed using a BBDuk Trimmer, and duplicated reads were removed. Host plant reads were kept or eliminated from the trimmed deduplicated reads by mapping them to the *Vitis* reference genome with medium-low sensitivity parameters. Small RNAs with and without grapevine reads were then assembled into contigs using Geneious (with medium-low sensitivity) and Velvet (with default parameters) assemblers. Next, blastn and tblastx were performed against the NCBI database of the reference viral and viroid genomes (refseq release date of 5 May 2022). Contigs with hits to plant viruses with an E-Value cutoff of <e^−5^ (or <e^−4^ in the analysis of contigs assembled by Velvet) were selected for further analysis. Trimmed reads were mapped to the reference genomes of the identified viruses, and the percentage of coverage of the reference genome was determined.

### 4.3. Validation of HTS Data

Validation was performed by RT-PCR. cDNA synthesis was carried out using 1 µg of total RNA of each sample, Random Hexamer, and RevertAid H Minus Reverse Transcriptase (EP0452, Thermo Fisher Scientific, Waltham, MA, USA) according to the manufacturer’s protocol. Then, PCR was performed with primers for the 18S rRNA gene and with primers for the grapevine viruses detected as a result of bioinformatics analysis (Supplementary Table S5). Primers for the validation of mycoviruses were designed based on the detected contigs using the NCBI Primer designing tool (Supplementary Table S5). PCR results were visualized by electrophoresis in 1.2% agarose gel. Amplicons were excised from the gel, extracted using a Cleanup Standard Kit for DNA (Evrogen, Russia) and sequenced using the Sanger method with two primers using a Big Dye Terminator v. 3.1 Cycle Sequencing Kit on an ABI PRIZM 3730 DNA Analyzer (Thermo Fisher Scientific, Waltham, MA, USA) according to the manufacturer’s protocol. The resulting nucleotide sequences were analyzed using Finch TV 1.4.0 software (*FinchTV*; Version 1.4.0; Geospiza, Inc.: Seattle, WA, USA, 2004–2012) and deposited in the GenBank (accession numbers available in Supplementary Table S8).

### 4.4. Phylogenetic and Recombination Analysis

For phylogenetic analysis, we used the complete genomes of viruses identified by HTS data analysis, together with complete genomes downloaded from the GenBank (Supplementary Table S9). Multiple sequence alignment was performed using the ClustalW method in BioEdit Sequence Alignment Editor v. 7.2.0 software [34]. Phylogenetic analysis was performed in the MegaX program using the neighbor-joining algorithm (1000 bootstrap replicates) [35]. The sequences of other virus species were used as an out-group. The clustering of identified virus isolates with representative isolates on the phylogenetic tree was a criterion for the determination of molecular groups (Supplementary Table S10). The resulting alignments were used to detect recombination events using the RDP v. 4.100 (Recombination Detection Program) [36] with default parameters. The conclusion regarding the presence of recombination points was made on the basis of the results obtained by the following methods: RDP, GENECONV, BOOTSCAN, MAXCHI, CHIMAERA, SISCAN, and 3SEQ. Recombination events meeting the criteria of being identified by four or more methods and having a *p*-value of 0.005 or less (*p* ≤ 0.05) were considered as positive.

## Figures and Tables

**Table 1:** Summary of bioinformatic analysis of HTS data and their validation on individual samples by targeted RT-PCR.

Sample Number	Viruses						Viroids	
	Clostero- Viridae	Betaflexi- Viridae			Tymo- Vidae			
	Ampelo- Virus	Tricho- Virus	Foveavirus		Macula-Virus	Marafi- Virus		
	GLRaV-3	GPGV	GVT	GRSPaV	GFkV	GRVFV	GYSVd-1	HSVd
593	−	+	+	+	+	−	−	+
594	+	+	−	+	−	+	+	+
596	+	+	−	+	−	+	+	+
598	−	+	−	−	+	+	−	+
599	−	+	−	+	+	−	−	+
601	−	+	−	+	+	−	−	+
603	−	+	−	+	+	−	−	+
605	−	+	−	+	−	−	−	+
HTS results	+	+	+	+	+	+	+	+

“+” positive result for RT-PCR (highlighted with green color) or HTS (highlighted with blue color) data analysis; “−“ negative result.

**Table 2:** Summary of mycovirus analysis.

Type of Analysis		ss(+) RNA									(−)ssRNA	RNA Satellites
		Mitoviridae						Hypo- Viridae	Narna- Viridae	Gamma- Flexi- Viridae	Unclassified (−) ssRNA Viruses	
		Mitovirus						Unclassified Hypo- Viridae	Narna- Virus	Myco- Flexi- Virus		
		Alternaria Arborescens Mitovirus 1	Botrytis Cinerea Mitovirus 1	Botrytis Cinerea Mitovirus 2	Botrytis Cinerea Mitovirus 3	Botrytis Cinerea Mitovirus 4	Sclero- Tinia Sclero- Tiorum Mitovirus 3	Botrytis Cinerea Hypo- Virus 1	Grape-Vine Associated Narna- Virus-1	Botrytis Virus F	Sclerotinia Sclerotiorum Negative-Stranded RNA Virus 3	Botrytis Cinerea Hypovirus 1 Satellite-like RNA
		AaMV-1	BcMV-1	BcMV-2	BcMV-3	BcMV-4	SsMV-3	BcHV-1	GaNV-1	BVF	SsNSRV-3	BcHV1 sat
		NC_030747.1	NC_011372.2	NC_028471.1	NC_028472.1	NC_028474.1	NC_028475.1	NC_037659.1	NC_028473.1	NC_002604.1	NC_026732.1	NC_037663.1
SPAdes+Contigs blastn		11	2	2	5	3	2	1	7	3	0	2
SPAdes+Contigs tblastx		11	2	2	5	3	2	1	7	3	6	2
Reads mapped to ref. seq		1120	345	167	126	321	231	57	593	64	0	38
Coverage of ref. seq, %		99.1	98.7	97.3	98.2	96.5	96.6	52.3	99.3	75.8	0	84.2
Pairwise identity to ref. seq, %		93.1	96.3	95.5	95.6	91.4	95.3	95.3	95.3	91.3	0	97.9
Validation by RT-PCR in grapevine samples	593	+	−	−	−	−	+	+	+	+	−	−
	594	+	−	−	−	−	−	−	−	−	−	−
	596	+	+	+	+	+	−	+	+	+	+	+
	598	−	−	−	+	−	−	−	−	−	−	−
	599	+	+	+	−	−	−	+	+	−	+	+
	601	−	−	+	−	−	−	+	−	−	−	−
	603	−	+	−	−	+	−	−	−	−	−	−
	605	+	+	−	+	−	−	−	−	−	−	−

“+”—virus was detected in the sample, “−“—virus was not detected in the sample. Sanger sequenced samples are highlighted with blue color.

## Data Availability

Representative sequences were deposited in GenBank under the accession numbers OL589426—OL589432, OL614977, OL961510, OL961512, OL961513, OL799308, OL799309, ON669263—ON669269, ON872514—ON872519, and ON714133—ON738340.

## Supplementary Materials

The following are available online at https://www.mdpi.com/article/10.3390/plants12183292/s1, Figure S1: Phylogenetic tree showing the distribution of nucleotide sequences of complete genome in Russian isolates of grapevine leafroll-associated virus 3. Figure S2: Phylogenetic tree showing the distribution of nucleotide sequences of coat protein genes in Russian isolates of grapevine leafroll-associated virus 3. Figure S3: Phylogenetic tree showing the distribution of nucleotide sequences of complete genome in Russian isolates of grapevine Pinot gris virus. Figure S4: Phylogenetic tree showing the distribution of nucleotide sequences of complete genome in Russian isolates of grapevine virus T. Figure S5: Phylogenetic tree showing the distribution of nucleotide sequences of complete genome in Russian isolates of grapevine yellow speckle viroid 1. Figure S6: Phylogenetic tree showing the distribution of nucleotide sequences of complete genome in Russian isolates of hop stunt viroid. Table S1: Grapevine samples and symptoms description (Rkatsiteli variety, Vitis vinifera Linne subsp. vinifera). Table S2: Initial statistics of rRNA-depleted RNAseq and sRNAseq, and de novo assembly. Table S3: Bioinformatic analysis of HTS results. Table S4: Analysis of complete genomes of viruses and viroids. Table S5: Primers used for the validation of HTS results. Table S6: Bioinformatics analysis of mycoviruses. Table S7: Validation of mycoviruses by RT-PCR. Table S8: List of sequences submitted to GenBank. Table S9: Initial data for phylogenetic and recombination analysis. Table S10: Representative virus isolates used for group definition. Supplementary analysis of mycoviruses contigs by NCBI Blastn tool. Supplementary validation of additional mycoviruses.
