# Supplementary material for: The First Virome of a Russian Vineyard
Source: Plants (Basel). 2023 Sep 18;12(18):3292. doi: 10.3390/plants12183292 (PMC10534617; doi:10.3390/plants12183292)
Supplement: Supplementary file 1 [file plants-12-03292-s001.zip › Supplementary Analysis of mycoviruses contigs by NCBI Blastn tool.pdf]

Supplementary materials

Analysis of mycoviruses contigs by NCBI Blastn tool

Phomopsis longicolla RNA virus 1

Contig 4525, length 1584 bp

Type of analysis: NCBI blastn, optimize for highly similar sequences (megablast)

Description	Scientific Name	Max Score	Total Score	Query Cover	E value	Per. Ident	Acc. Len	Accession
Sclerotinia sclerotiorum hypovirus 7 satellite-like RNA isolate GB375-3 hypothetical protein gene, complete cds	Sclerotinia sclerotiorum hypovirus 7 satellite-like RNA	1568	5545	89%	0.0	97.18%	4316	MW454886.1
Botrytis cinerea hypovirus 1 satellite-like RNA, complete genome	Botrytis cinerea hypovirus 1 satellite-like RNA	1563	5517	89%	0.0	97.07%	3960	NC_037663.1

Contig 12041, length 719 bp

Type of analysis: NCBI blastn, optimize for highly similar sequences (megablast)

Description	Scientific Name	Max Score	Total Score	Query Cover	E value	Per. Ident	Acc. Len	Accession

Description	Scientific Name	Max Score	Total Score	Query Cover	E value	Per. Ident	Acc. Len	Accession
Erysiphe necator associated ourmia-like virus 121 isolate PMS8_191, complete genome	Erysiphe necator associated ourmia-like virus 121	1013	1013	100%	0.0	92.07%	2346	MN611648.1
Plasmopara viticola lesion associated ourmia-like virus 85 isolate DMG-C_28820 RNA dependent RNA polymerase gene, complete cds	Plasmopara viticola lesion associated ourmia-like virus 85	985	985	100%	0.0	91.38%	3060	MN532672.1

Magnaporte oryzae ourmia-like virus

Contig 16448, length 548 bp

Type of analysis: NCBI blastn, optimize for highly similar sequences (megablast)

Description	Scientific Name	Max Score	Total Score	Query Cover	E value	Per. Ident	Acc. Len	Accession
Plasmopara viticola lesion associated ourmia-like virus 6 isolate DMG-B_8483 RNA dependent RNA polymerase gene, complete cds	Plasmopara viticola lesion associated ourmia-like virus 6	721	721	99%	0.0	90.48%	2697	MN532593.1

Gremmeniella abietina RNA virus MS1

Contig 18015, length 511 bp

Type of analysis: NCBI blastn, optimize for highly similar sequences (megablast)

Description	Scientific Name	Max Score	Total Score	Query Cover	E value	Per. Ident	Acc. Len	Accession
Botrytis cinerea partitivirus 3 isolate BCS4_DN10017 segment RNA1, complete sequence	Botrytis cinerea partitivirus 3	869	869	99%	0.0	97.45%	1762	MN954884.1
Sclerotinia sclerotiorum partitivirus 3 isolate SsPV3 RNA-dependent RNA polymerase gene, partial cds	Sclerotinia sclerotiorum partitivirus 3	857	857	99%	0.0	97.05%	1769	MF444214.1

Fusarium redolens polymycovirus 1

Contig 7922, legnht 1,050 bp

Type of analysis: More dissimilar sequences (discontiguous megablast), similar sequences (blastn)

Description	Scientific Name	Max Score	Total Score	Query Cover	E value	Per. Ident	Acc. Len	Accession
Fusarium redolens polymycovirus 1 isolate FRPV.A63-1 segment RNA 6, complete sequence	Fusarium redolens polymycovirus 1	137	137	44%	6e-27	67.51%	1015	NC_055276.1

Fusarium poae negative-stranded virus 2

Contig 15923, length 563 bp

Type of analysis: More dissimilar sequences (discontiguous megablast), similar sequences (blastn)

Description	Scientific Name	Max Score	Total Score	Query Cover	E value	Per. Ident	Acc. Len	Accession
Coniothyrium diplodiella negative-stranded RNA virus 1 isolate CREA-VE-6SY1, complete sequence	Coniothyrium diplodiella negative-stranded RNA virus 1	219	219	93%	3e-52	70.40%	6519	MK584854.1
Plasmopara viticola lesion associated mycobunyavirales-like virus 9 isolate DMG-E_DN19612 segment RNA1 RNA-dependent RNA polymerase gene, complete cds	Plasmopara viticola lesion associated mycobunyavirales-like virus 9	212	212	98%	5e-50	68.89%	6499	MN585282.1

Fusarium poae narnavirus 2

Contig 16649, length 543 bp

Type of analysis: NCBI blastn, optimize for highly similar sequences (megablast)

Description	Scientific Name	Max Score	Total Score	Query Cover	E value	Per. Ident	Acc. Len	Accession
Neofusicoccum parvum narnavirus 2 isolate CREA-VE-9AS3, complete sequence	Neofusicoccum parvum narnavirus 2	953	953	100%	0.0	98.34%	2026	MK584834.1
Erysiphe necator associated narnavirus 30 isolate PMS3_DN12264, complete genome	Erysiphe necator associated narnavirus 30	237	237	64%	1e-57	79.22%	2154	MN605443.1
Erysiphe necator associated narnavirus 5 isolate PMS-18_DN51913_ RdRp gene, complete cds	Erysiphe necator associated narnavirus 5	231	231	68%	6e-56	78.48%	2043	MN557023.1
Cryphonectria naterciae splipalmivirus 1 C0614 RNA, segment RNA1, complete sequence	Cryphonectria naterciae splipalmivirus 1	213	213	63%	2e-50	78.19%	2172	LC634419.1

Description	Scientific Name	Max Score	Total Score	Query Cover	E value	Per. Ident	Acc. Len	Accession
Aspergillus fumigatus narnavirus 2 J-YC-3 genomic RNA, segment 1, complete sequence	Aspergillus fumigatus narnavirus 2	99.0	99.0	14%	6e-16	89.61%	2008	LC553693.1

Fusarium poae mitovirus 4

Contig 1983, length 2,392 bp

Type of analysis: NCBI blastn, optimize for highly similar sequences (megablast)

Description	Scientific Name	Max Score	Total Score	Query Cover	E value	Per. Ident	Acc. Len	Accession
Botrytis cinerea mitovirus 6 isolate BCI1_DN10328 RNA-dependent RNA polymerase gene, complete cds	Botrytis cinerea mitovirus 6	4019	4019	100%	0.0	96.99%	2519	MN625252.1

Fusarium poae mitovirus 3

Contig 17879, length 514 bp

Type of analysis: NCBI blastn, optimize for highly similar sequences (megablast)

Description	Scientific Name	Max Score	Total Score	Query Cover	E value	Per. Ident	Acc. Len	Accession
Fusarium asiaticum mitovirus 2 strain GS-6 RNA-dependent RNA polymerase gene, complete cds	Fusarium asiaticum mitovirus 2	767	767	100%	0.0	93.59%	2636	MZ969052.1
Soybean leaf-associated mitovirus 5 isolate SaMitV5-1, partial sequence	Soybean leaf-associated mitovirus 5	706	706	100%	0.0	91.46%	2665	KT598237.1
Fusarium sambucinum mitovirus 1 Fs2242m1 RdRp	Fusarium sambucinum	555	555	99%	3e-153	86.16%	2650	LC596825.1

Description	Scientific Name	Max Score	Total Score	Query Cover	E value	Per. Ident	Acc. Len	Accession
gene for RNA dependent RNA polymerase, complete cds	mitovirus 1							
Plasmopara viticola lesion associated mitovirus 24 isolate DMG-G_Contig5 RNA-dependent RNA polymerase gene, complete cds	Plasmopara viticola lesion associated mitovirus 24	298	298	97%	5e-76	77.67%	2651	MN539785.1

Contig 14563, length 605 bp

Type of analysis: More dissimilar sequences (discontiguous megablast), similar sequences (blastn)

Description	Scientific Name	Max Score	Total Score	Query Cover	E value	Per. Ident	Acc. Len	Accession
Plasmopara viticola lesion associated mitovirus 24 isolate DMG-G_Contig5 RNA-dependent RNA polymerase gene, complete cds	Plasmopara viticola lesion associated mitovirus 24	331	331	100%	8e-86	72.62%	2651	MN539785.1
Fusarium mangiferae mitovirus 3 isolate FmMV3/SP1, complete genome	Fusarium mangiferae mitovirus 3	242	242	94%	1e-58	69.88%	2637	MZ493903.1

Contig 12969, length 670 bp

Type of analysis: NCBI blastn, optimize for highly similar sequences (megablast)

Description	Scientific Name	Max Score	Total Score	Query Cover	E value	Per. Ident	Acc. Len	Accession
Plasmopara viticola lesion associated mitovirus 24 isolate DMG-G_Contig5 RNA-dependent RNA polymerase gene, complete cds	Plasmopara viticola lesion associated mitovirus 24	364	364	89%	7e-96	77.81%	2651	MN539785.1

Contig 9484, length 898 bp

Type of analysis: NCBI blastn, optimize for highly similar sequences (megablast)

Description	Scientific Name	Max Score	Total Score	Query Cover	E value	Per. Ident	Acc. Len	Accession
Plasmopara viticola lesion associated mitovirus 24 isolate DMG-G_Contig5 RNA-dependent RNA polymerase gene, complete cds	Plasmopara viticola lesion associated mitovirus 24	795	795	100%	0.0	82.69%	2651	MN539785.1

Cladosporium fulvum T-1 virus

Contig 14847, length 596 bp

Type of analysis: More dissimilar sequences (discontiguous megablast), similar sequences (blastn)

Description	Scientific Name	Max Score	Total Score	Query Cover	E value	Per. Ident	Acc. Len	Accession
Cladosporium fulvum T-1 virus LTR-retrotransposon encoding homologues to retroviral gag, pol, and env genes	Cladosporium fulvum T-1 virus	310	310	68%	9e-79	66.20%	7396	NC_043491.1

Contig 11315, length 763 bp

Type of analysis: More dissimilar sequences (discontiguous megablast), similar sequences (blastn)

Description	Scientific Name	Max Score	Total Score	Query Cover	E value	Per. Ident	Acc. Len	Accession
Cladosporium fulvum T-1 virus LTR-retrotransposon encoding homologues to retroviral gag, pol, and env genes	Cladosporium fulvum T-1 virus	266	266	79%	4e-66	69.89%	7396	NC_043491.1

Contig 8424, length 996 bp

Type of analysis: More dissimilar sequences (discontiguous megablast), similar sequences (blastn)

Description	Scientific Name	Max Score	Total Score	Query Cover	E value	Per. Ident	Acc. Len	Accession
Cladosporium fulvum T-1 virus	Cladosporium fulvum T-1 virus	292	292	75%	1e-73	70.20%	7396	NC_043491.1

Contig 2619, length 2109 bp

Type of analysis: More dissimilar sequences (discontiguous megablast), similar sequences (blastn)

Description	Scientific Name	Max Score	Total Score	Query Cover	E value	Per. Ident	Acc. Len	Accession
Cladosporium fulvum T-1 virus LTR-retrotransposon encoding homologues to retroviral gag, pol, and env genes	Cladosporium fulvum T-1 virus	310	310	68%	9e-79	66.20%	7396	NC_043491.1

Botrytis virus F

Contig 19266, length 487 bp

Type of analysis: NCBI blastn, optimize for highly similar sequences (megablast)

Description	Scientific Name	Max Score	Total Score	Query Cover	E value	Per. Ident	Acc. Len	Accession
Botrytis virus F RdRp gene for RNA dependent RNA polymerase, isolate HAZ3-8A	Botrytis virus F	712	712	98%	0.0	93.35%	6814	LN827953.1
Botrytis virus F, complete genome	Botrytis virus F	575	575	98%	2e-159	88.31%	6827	AF238884.1

Contig 19177, length 489 bp

Type of analysis: NCBI blastn, optimize for highly similar sequences (megablast)

Description	Scientific Name	Max Score	Total Score	Query Cover	E value	Per. Ident	Acc. Len	Accession
Botrytis virus F RdRp gene for RNA dependent RNA polymerase, isolate HAZ3-8A	Botrytis virus F	675	675	99%	0.0	91.60%	6814	LN827953.1
Botrytis virus F, complete genome	Botrytis virus F	586	586	99%	9e-163	88.32%	6827	AF238884.1

Contig 15190, length 585 bp

Type of analysis: NCBI blastn, optimize for highly similar sequences (megablast)

Description	Scientific Name	Max Score	Total Score	Query Cover	E value	Per. Ident	Acc. Len	Accession
Botrytis virus F RdRp gene for RNA dependent RNA polymerase, isolate HAZ3-8A	Botrytis virus F	802	802	100%	0.0	91.45%	6814	LN827953.1
Botrytis virus F, complete genome	Botrytis virus F	625	625	100%	2e-174	85.98%	6827	AF238884.1

Botrytis cinerea mitovirus 4

Contig 13187, length 661 bp

Type of analysis: NCBI blastn, optimize for highly similar sequences (megablast)

Description	Scientific Name	Max Score	Total Score	Query Cover	E value	Per. Ident	Acc. Len	Accession
Botrytis cinerea mitovirus 5 isolate BCS17_DN132, complete genome	Botrytis cinerea mitovirus 5	989	989	100%	0.0	93.65%	2721	MN617167.1
Botrytis cinerea mitovirus 4 RdRp gene for RNA dependent RNA polymerase, isolate HAZ3-4	Botrytis cinerea mitovirus 4	983	983	100%	0.0	93.49%	2768	LN827947.1

Description	Scientific Name	Max Score	Total Score	Query Cover	E value	Per. Ident	Acc. Len	Accession
Sclerotinia sclerotiorum mitovirus 36, partial genome	Sclerotinia sclerotiorum mitovirus 36	955	955	99%	0.0	92.99%	2732	MT646380.1
Sclerotinia nivalis mitovirus 1 isolate SsSn-1.m1, complete genome	Sclerotinia nivalis mitovirus 1	889	889	99%	0.0	91.15%	2720	KT365895.1
Botrytis cinerea mitovirus 4 isolate BCS16_23, complete genome	Botrytis cinerea mitovirus 4	889	889	100%	0.0	90.94%	2709	MN954875.1
Botrytis cinerea mitovirus 7 isolate BCI5_DN3722, complete genome	Botrytis cinerea mitovirus 7	662	662	99%	0.0	84.93%	2705	MN617168.1
Sclerotinia sclerotiorum mitovirus 4 RNA-dependent RNA polymerase gene, complete cds	Sclerotinia sclerotiorum mitovirus 4	623	623	99%	1e-173	83.96%	2744	JX401538.1
Sclerotinia sclerotiorum mitovirus 4 isolate BCS16_24, complete genome	Sclerotinia sclerotiorum mitovirus 4	595	595	99%	2e-165	83.21%	2737	MN954877.1
Sclerotinia sclerotiorum mitovirus 4-A isolate SsMV4-A RNA-dependent RNA polymerase gene, complete cds	Sclerotinia sclerotiorum mitovirus 4-A	573	573	100%	1e-158	82.43%	2737	MF444237.1
Sclerotinia sclerotiorum mitovirus 4, complete sequence	Sclerotinia sclerotiorum mitovirus 4	501	501	99%	5e-137	80.55%	2752	KT962974.1
Grapevine associated narnavirus-1 isolate Ctg501 putative RNA-dependent RNA polymerase gene, complete cds	Grapevine associated narnavirus-1	411	411	64%	8e-110	84.06%	622	GU108590.1
Sclerotinia sclerotiorum mitovirus 39, partial genome	Sclerotinia sclerotiorum mitovirus 39	390	390	99%	1e-103	77.74%	2614	MT646411.1

Contig 3592, length 1812 bp

Type of analysis: NCBI blastn, optimize for highly similar sequences (megablast)

Description	Scientific Name	Max Score	Total Score	Query Cover	E value	Per. Ident	Acc. Len	Accession
Sclerotinia sclerotiorum mitovirus 4 RNA-dependent RNA polymerase gene, complete cds	Sclerotinia sclerotiorum mitovirus 4	3031	3031	100%	0.0	96.85%	2744	JX401538.1
Sclerotinia sclerotiorum mitovirus 4 isolate BCS16_24, complete genome	Sclerotinia sclerotiorum mitovirus 4	2964	2964	100%	0.0	96.19%	2737	MN954877.1
Sclerotinia sclerotiorum mitovirus 4-A isolate SsMV4-A RNA-dependent RNA polymerase gene, complete cds	Sclerotinia sclerotiorum mitovirus 4-A	2704	2704	100%	0.0	93.60%	2737	MF444237.1
Sclerotinia sclerotiorum mitovirus 4, complete sequence	Sclerotinia sclerotiorum mitovirus 4	2671	2671	100%	0.0	93.27%	2752	KT962974.1
Botrytis cinerea mitovirus 5 isolate BCS17_DN132, complete genome	Botrytis cinerea mitovirus 5	2455	2455	100%	0.0	91.13%	2721	MN617167.1
Sclerotinia sclerotiorum mitovirus 4 isolate Landkreis Rostock_17 putative RNA-dependent RNA polymerase gene, partial cds	Sclerotinia sclerotiorum mitovirus 4	1674	1674	56%	0.0	95.99%	1023	MN399689.1
Sclerotinia sclerotiorum mitovirus 39, partial genome	Sclerotinia sclerotiorum mitovirus 39	1544	1544	92%	0.0	83.35%	2614	MT646411.1
Sclerotinia sclerotiorum mitovirus 36, partial genome	Sclerotinia sclerotiorum mitovirus 36	1461	1461	96%	0.0	81.88%	2732	MT646380.1
Botrytis cinerea mitovirus 7 isolate BCI5_DN3722, complete genome	Botrytis cinerea mitovirus 7	1452	1452	96%	0.0	81.81%	2705	MN617168.1
Botrytis cinerea mitovirus 4 isolate BCS16_23, complete genome	Botrytis cinerea mitovirus 4	1450	1450	96%	0.0	81.74%	2709	MN954875.1
Sclerotinia nivalis mitovirus 1 isolate SsSn-1.m1, complete genome	Sclerotinia nivalis mitovirus 1	1447	1447	96%	0.0	81.78%	2720	KT365895.1
Botrytis cinerea mitovirus 4 RdRp gene for RNA dependent RNA polymerase, isolate HAZ3-4	Botrytis cinerea mitovirus 4	1411	1411	96%	0.0	81.41%	2768	LN827947.1

Contig 1479, length 2717 bp

Type of analysis: NCBI blastn, optimize for highly similar sequences (megablast)

Description	Scientific Name	Max Score	Total Score	Query Cover	E value	Per. Ident	Acc. Len	Accession
Sclerotinia sclerotiorum mitovirus 4 RNA-dependent RNA polymerase gene, complete cds	Sclerotinia sclerotiorum mitovirus 4	4462	4462	100%	0.0	96.36%	2744	JX401538.1
Sclerotinia sclerotiorum mitovirus 4 isolate BCS16_24, complete genome	Sclerotinia sclerotiorum mitovirus 4	4458	4458	100%	0.0	96.28%	2737	MN954877.1
Sclerotinia sclerotiorum mitovirus 4, complete sequence	Sclerotinia sclerotiorum mitovirus 4	4000	4000	100%	0.0	93.24%	2752	KT962974.1
Sclerotinia sclerotiorum mitovirus 4-A isolate SsMV4-A RNA-dependent RNA polymerase gene, complete cds	Sclerotinia sclerotiorum mitovirus 4-A	3925	3925	100%	0.0	92.76%	2737	MF444237.1
Botrytis cinerea mitovirus 4 isolate BCS16_23, complete genome	Botrytis cinerea mitovirus 4	2872	2872	97%	0.0	86.27%	2709	MN954875.1
Botrytis cinerea mitovirus 5 isolate BCS17_DN132, complete genome	Botrytis cinerea mitovirus 5	2856	2856	91%	0.0	87.52%	2721	MN617167.1
Sclerotinia sclerotiorum mitovirus 36, partial genome	Sclerotinia sclerotiorum mitovirus 36	1925	1925	89%	0.0	81.21%	2732	MT646380.1
Sclerotinia sclerotiorum mitovirus 39, partial genome	Sclerotinia sclerotiorum mitovirus 39	1917	1917	84%	0.0	81.83%	2614	MT646411.1
Botrytis cinerea mitovirus 4 RdRp gene for RNA dependent RNA polymerase, isolate HAZ3-4	Botrytis cinerea mitovirus 4	1908	1908	88%	0.0	81.08%	2768	LN827947.1
Botrytis cinerea mitovirus 7 isolate BCI5_DN3722, complete genome	Botrytis cinerea mitovirus 7	1853	1853	88%	0.0	80.69%	2705	MN617168.1
Sclerotinia nivalis mitovirus 1 isolate SsSn-1.m1, complete genome	Sclerotinia nivalis mitovirus 1	1847	1847	88%	0.0	80.63%	2720	KT365895.1

Botrytis cinerea mitovirus 3

Contig 18936, length 493 bp

Type of analysis: NCBI blastn, optimize for highly similar sequences (megablast)

Description	Scientific Name	Max Score	Total Score	Query Cover	E value	Per. Ident	Acc. Len	Accession
Botrytis cinerea mitovirus 3 isolate BCS16_12, complete genome	Botrytis cinerea mitovirus 3	701	701	100%	0.0	92.31%	2977	MN617166.1
Botrytis cinerea mitovirus 3 RdRp gene for RNA dependent RNA polymerase, isolate HAZ1-3	Botrytis cinerea mitovirus 3	695	695	100%	0.0	92.11%	2922	LN827942.1
Botrytis cinerea mitovirus 3 RdRp gene for RNA dependent RNA polymerase, isolate HAZ3-3	Botrytis cinerea mitovirus 3	656	656	100%	0.0	90.67%	2930	LN827946.1
Sclerotinia sclerotiorum mitovirus 35, partial genome	Sclerotinia sclerotiorum mitovirus 35	601	601	97%	3e-167	89.23%	2879	MT646369.1

Contig 18362, length 504 bp

Type of analysis: NCBI blastn, optimize for highly similar sequences (megablast)

Description	Scientific Name	Max Score	Total Score	Query Cover	E value	Per. Ident	Acc. Len	Accession

Description	Scientific Name	Max Score	Total Score	Query Cover	E value	Per. Ident	Acc. Len	Accession
Botrytis cinerea mitovirus 3 RdRp gene for RNA dependent RNA polymerase, isolate HAZ1-3	Botrytis cinerea mitovirus 3	780	780	100%	0.0	94.67%	2922	LN827942.1
Botrytis cinerea mitovirus 3 RdRp gene for RNA dependent RNA polymerase, isolate HAZ3-3	Botrytis cinerea mitovirus 3	763	820	100%	0.0	94.94%	2930	LN827946.1
Botrytis cinerea mitovirus 3 isolate BCS16_12, complete genome	Botrytis cinerea mitovirus 3	739	739	100%	0.0	93.27%	2977	MN617166.1
Sclerotinia sclerotiorum mitovirus 35, partial genome	Sclerotinia sclerotiorum mitovirus 35	680	680	99%	0.0	91.29%	2879	MT646369.1

Contig 15743, length 568 bp

Type of analysis: NCBI blastn, optimize for highly similar sequences (megablast)

Description	Scientific Name	Max Score	Total Score	Query Cover	E value	Per. Ident	Acc. Len	Accession
Botrytis cinerea mitovirus 3 RdRp gene for RNA dependent RNA polymerase, isolate HAZ3-3	Botrytis cinerea mitovirus 3	880	880	99%	0.0	94.71%	2930	LN827946.1
Botrytis cinerea mitovirus 3 isolate BCS16_12, complete genome	Botrytis cinerea mitovirus 3	857	857	99%	0.0	94.01%	2977	MN617166.1
Botrytis cinerea mitovirus 3 RdRp gene for RNA dependent RNA polymerase, isolate HAZ1-3	Botrytis cinerea mitovirus 3	852	852	99%	0.0	93.84%	2922	LN827942.1
Sclerotinia sclerotiorum mitovirus 35, partial genome	Sclerotinia sclerotiorum mitovirus 35	800	800	99%	0.0	92.15%	2879	MT646369.1

Contig 11674, length 740 bp

Type of analysis: NCBI blastn, optimize for highly similar sequences (megablast)

Description	Scientific Name	Max Score	Total Score	Query Cover	E value	Per. Ident	Acc. Len	Accession
Botrytis cinerea mitovirus 3 RdRp gene for RNA dependent RNA polymerase, isolate HAZ1-3	Botrytis cinerea mitovirus 3	1031	1031	100%	0.0	91.82%	2922	LN827942.1
Botrytis cinerea mitovirus 3 isolate BCS16_12, complete genome	Botrytis cinerea mitovirus 3	1020	1020	99%	0.0	91.87%	2977	MN617166.1
Botrytis cinerea mitovirus 3 RdRp gene for RNA dependent RNA polymerase, isolate HAZ3-3	Botrytis cinerea mitovirus 3	970	970	96%	0.0	91.14%	2930	LN827946.1
Sclerotinia sclerotiorum mitovirus 35, partial genome	Sclerotinia sclerotiorum mitovirus 35	907	907	98%	0.0	89.18%	2879	MT646369.1

Contig 4076, length 1683 bp

Type of analysis: NCBI blastn, optimize for highly similar sequences (megablast)

Description	Scientific Name	Max Score	Total Score	Query Cover	E value	Per. Ident	Acc. Len	Accession
Botrytis cinerea mitovirus 3 isolate BCS16_12, complete genome	Botrytis cinerea mitovirus 3	2519	2519	99%	0.0	93.70%	2977	MN617166.1
Botrytis cinerea mitovirus 3 RdRp gene for RNA dependent RNA polymerase, isolate HAZ1-3	Botrytis cinerea mitovirus 3	2440	2440	99%	0.0	92.87%	2922	LN827942.1
Botrytis cinerea mitovirus 3 RdRp gene for RNA dependent RNA polymerase, isolate HAZ3-3	Botrytis cinerea mitovirus 3	2433	2433	100%	0.0	92.76%	2930	LN827946.1
Sclerotinia sclerotiorum mitovirus 35, partial genome	Sclerotinia sclerotiorum mitovirus 35	2287	2287	99%	0.0	91.20%	2879	MT646369.1

Botrytis cinerea mitovirus 2

Contig 11663, length 741 bp

Type of analysis: NCBI blastn, optimize for highly similar sequences (megablast)

Description	Scientific Name	Max Score	Total Score	Query Cover	E value	Per. Ident	Acc. Len	Accession
Botrytis cinerea mitovirus 2 RdRp gene for RNA dependent RNA polymerase, isolate HAZ1-2	Botrytis cinerea mitovirus 2	1297	1297	100%	0.0	98.25%	2497	LN827941.1
Botrytis cinerea mitovirus 2 RdRp gene for RNA dependent RNA polymerase, isolate HAZ3-2	Botrytis cinerea mitovirus 2	1175	1175	100%	0.0	95.28%	2496	LN827945.1
Botrytis cinerea mitovirus 2 isolate BCS8_13, complete genome	Botrytis cinerea mitovirus 2	1175	1175	100%	0.0	95.28%	2502	MN617165.1

Contig 2721, length 2072 bp

Type of analysis: NCBI blastn, optimize for highly similar sequences (megablast)

Description	Scientific Name	Max Score	Total Score	Query Cover	E value	Per. Ident	Acc. Len	Accession
Botrytis cinerea mitovirus 2 isolate BCS8_13, complete genome	Botrytis cinerea mitovirus 2	3168	3168	99%	0.0	94.26%	2502	MN617165.1
Botrytis cinerea mitovirus 2 RdRp gene for RNA dependent RNA polymerase, isolate HAZ1-2	Botrytis cinerea mitovirus 2	2887	2887	100%	0.0	91.95%	2497	LN827941.1
Botrytis cinerea mitovirus 2 RdRp gene for RNA dependent RNA polymerase, isolate HAZ3-2	Botrytis cinerea mitovirus 2	2832	2832	100%	0.0	91.47%	2496	LN827945.1

Botrytis cinerea mitovirus 1

Contig 1406, length 2767 bp

Type of analysis: NCBI blastn, optimize for highly similar sequences (megablast)

Description	Scientific Name	Max Score	Total Score	Query Cover	E value	Per. Ident	Acc. Len	Accession
Botrytis cinerea mitovirus 1 isolate BCS1_DN4879 RNA-dependent RNA polymerase gene, complete cds	Botrytis cinerea mitovirus 1	4638	4638	100%	0.0	96.93%	2788	MT119677.1
Botrytis cinerea mitovirus 1 RdRp gene for RNA dependent RNA polymerase, isolate HAZ1-1	Botrytis cinerea mitovirus 1	4632	4755	100%	0.0	96.89%	2873	LN827940.1
Botrytis cinerea mitovirus 1 RNA-dependent RNA polymerase (RDRP) gene, complete cds	Botrytis cinerea mitovirus 1	4573	4573	100%	0.0	96.50%	2804	EF580100.3
Botrytis cinerea mitovirus 1 RdRp gene for RNA dependent RNA polymerase, isolate HAZ3-1	Botrytis cinerea mitovirus 1	4377	4377	100%	0.0	95.23%	2820	LN827944.1

Contig 1407, length 2767 bp

Type of analysis: NCBI blastn, optimize for highly similar sequences (megablast)

Description	Scientific Name	Max Score	Total Score	Query Cover	E value	Per. Ident	Acc. Len	Accession
Botrytis cinerea mitovirus 1 isolate BCS1_DN4879 RNA-dependent RNA polymerase gene, complete cds	Botrytis cinerea mitovirus 1	4721	4721	100%	0.0	97.47%	2788	MT119677.1
Botrytis cinerea mitovirus 1 RdRp gene for RNA dependent RNA polymerase, isolate HAZ1-1	Botrytis cinerea mitovirus 1	4577	4700	100%	0.0	96.53%	2873	LN827940.1
Botrytis cinerea mitovirus 1 RNA-dependent RNA polymerase (RDRP) gene, complete cds	Botrytis cinerea mitovirus 1	4501	4501	100%	0.0	96.03%	2804	EF580100.3
Botrytis cinerea mitovirus 1 RdRp gene for RNA dependent RNA polymerase, isolate HAZ3-1	Botrytis cinerea mitovirus 1	4366	4366	100%	0.0	95.16%	2820	LN827944.1

Botrytis cinerea hypovirus 1

Contig 19526, length 483 bp

Type of analysis: NCBI blastn, optimize for highly similar sequences (megablast)

Description	Scientific Name	Max Score	Total Score	Query Cover	E value	Per. Ident	Acc. Len	Accession
Sclerotinia sclerotiorum hypovirus 7 isolate GB375-2 polyprotein gene, complete cds	Sclerotinia sclerotiorum hypovirus 7	876	876	100%	0.0	99.38%	10218	MW454885.1
Botrytis cinerea hypovirus 1 strain BcHv1.HBstr-470, complete genome	Botrytis cinerea hypovirus 1	826	826	100%	0.0	97.52%	10250	MH347277.1
Sclerotinia sclerotiorum hypovirus 3 isolate SsHV3S1 polyprotein gene, partial cds	Sclerotinia sclerotiorum hypovirus 3	826	826	100%	0.0	97.52%	4624	MF444222.1
Botrytis cinerea hypovirus 1, complete genome	Botrytis cinerea hypovirus 1	826	826	100%	0.0	97.52%	10252	NC_037659.1
Sclerotinia sclerotiorum hypovirus 7, partial genome	Sclerotinia sclerotiorum hypovirus 7	815	815	100%	0.0	97.10%	10208	MT646394.1

Botrytis cinerea hypo virus1 sattelite-like RNA

Contig 15149, length 586 bp

Type of analysis: NCBI blastn, optimize for highly similar sequences (megablast)

Description	Scientific Name	Max Score	Total Score	Query Cover	E value	Per. Ident	Acc. Len	Accession
Sclerotinia sclerotiorum hypovirus 7 satellite-like RNA isolate GB375-3 hypothetical protein gene, complete cds	Sclerotinia sclerotiorum hypovirus 7 satellite-like RNA	1016	1016	100%	0.0	97.95%	4316	MW454886.1
Botrytis cinerea hypovirus 1 satellite-like RNA, complete genome	Botrytis cinerea hypovirus 1 satellite-like RNA	1011	1011	100%	0.0	97.78%	3960	NC_037663.1
Sclerotinia sclerotiorum hypovirus 7 satellite-like RNA, partial genome	Sclerotinia sclerotiorum hypovirus 7 satellite-like RNA	1000	1000	100%	0.0	97.44%	3904	MT646409.1

Contig 12853, length 676 bp

Type of analysis: NCBI blastn, optimize for highly similar sequences (megablast)

Description	Scientific Name	Max Score	Total Score	Query Cover	E value	Per. Ident	Acc. Len	Accession
Sclerotinia sclerotiorum hypovirus 7 satellite-like RNA, partial genome	Sclerotinia sclerotiorum hypovirus 7 satellite-like RNA	1170	1170	99%	0.0	97.93%	3904	MT646409.1
Botrytis cinerea hypovirus 1 satellite-like RNA, complete genome	Botrytis cinerea hypovirus 1 satellite-like RNA	1164	1164	99%	0.0	97.78%	3960	NC_037663.1
Sclerotinia sclerotiorum hypovirus 7 satellite-like RNA isolate GB375-3 hypothetical protein gene, complete cds	Sclerotinia sclerotiorum hypovirus 7 satellite-like RNA	1127	1127	96%	0.0	97.71%	4316	MW454886.1
Botrytis cinerea hypovirus 1 satellite-like RNA-S, complete genome	Botrytis cinerea hypovirus 1 satellite-like RNA-S	832	832	71%	0.0	97.53%	1413	MG554637.1

Alternaria arborescens mitovirus 1

Contig 20862, length 386 bp

Type of analysis: NCBI blastn, optimize for highly similar sequences (megablast)

Description	Scientific Name	Max Score	Total Score	Query Cover	E value	Per. Ident	Acc. Len	Accession
Alternaria arborescens mitovirus 1 isolate PMS8_29, complete genome	Alternaria arborescens mitovirus 1	619	619	100%	7e-173	95.60%	2483	MN599397.1
Alternaria arborescens mitovirus 1 genomic RNA, complete genome	Alternaria arborescens mitovirus 1	601	601	99%	3e-167	94.81%	2506	LC145036.1
Soybean leaf-associated mitovirus 2 isolate SaMitV2-1, partial sequence	Soybean leaf-associated mitovirus 2	475	475	79%	2e-129	94.77%	2477	KT598239.1

Contig 20388, length 463 bp

Type of analysis: NCBI blastn, optimize for highly similar sequences (megablast)

Description	Scientific Name	Max Score	Total Score	Query Cover	E value	Per. Ident	Acc. Len	Accession
Alternaria arborescens mitovirus 1 isolate PMS8_29, complete genome	Alternaria arborescens mitovirus 1	756	756	100%	0.0	96.12%	2483	MN599397.1
Alternaria arborescens mitovirus 1 genomic RNA, complete genome	Alternaria arborescens mitovirus 1	667	667	100%	0.0	92.66%	2506	LC145036.1
Soybean leaf-associated mitovirus 2 isolate SaMitV2-1, partial sequence	Soybean leaf-associated mitovirus 2	507	507	99%	7e-139	86.67%	2477	KT598239.1
Uncultured mitochondrial RNA virus genomic RNA containing putative RdRp, contig 162Hv2_454_2_1	uncultured mitochondrial RNA virus	460	460	100%	6e-125	84.58%	698	HE579567.1

Contig 17637, length 519 bp

Type of analysis: NCBI blastn, optimize for highly similar sequences (megablast)

Description	Scientific Name	Max Score	Total Score	Query Cover	E value	Per. Ident	Acc. Len	Accession
Alternaria arborescens mitovirus 1 genomic RNA, complete genome	Alternaria arborescens mitovirus 1	737	737	100%	0.0	92.31%	2506	LC145036.1
Alternaria arborescens mitovirus 1 isolate PMS8_29, complete genome	Alternaria arborescens mitovirus 1	688	688	99%	0.0	90.70%	2483	MN599397.1
Soybean leaf-associated mitovirus 2 isolate SaMitV2-1, partial sequence	Soybean leaf-associated mitovirus 2	660	660	100%	0.0	89.67%	2477	KT598239.1

Contig 15296, length 581 bp

Type of analysis: NCBI blastn, optimize for highly similar sequences (megablast)

Description	Scientific Name	Max Score	Total Score	Query Cover	E value	Per. Ident	Acc. Len	Accession
Alternaria arborescens mitovirus 1 genomic RNA, complete genome	Alternaria arborescens mitovirus 1	926	926	99%	0.0	95.52%	2506	LC145036.1
Alternaria arborescens mitovirus 1 isolate PMS8_29, complete genome	Alternaria arborescens mitovirus 1	922	922	97%	0.0	95.81%	2483	MN599397.1

Contig 14791, length 597 bp

Type of analysis: NCBI blastn, optimize for highly similar sequences (megablast)

Description	Scientific Name	Max Score	Total Score	Query Cover	E value	Per. Ident	Acc. Len	Accession
Alternaria arborescens mitovirus 1 genomic RNA, complete genome	Alternaria arborescens mitovirus 1	983	983	99%	0.0	96.48%	2506	LC145036.1
Alternaria arborescens mitovirus 1 isolate PMS8_29, complete genome	Alternaria arborescens mitovirus 1	952	952	97%	0.0	95.93%	2483	MN599397.1

Contig 13918, length 630 bp

Type of analysis: NCBI blastn, optimize for highly similar sequences (megablast)

Description	Scientific Name	Max Score	Total Score	Query Cover	E value	Per. Ident	Acc. Len	Accession
Alternaria arborescens mitovirus 1 genomic RNA, complete genome	Alternaria arborescens mitovirus 1	1026	1026	100%	0.0	96.03%	2506	LC145036.1

Description	Scientific Name	Max Score	Total Score	Query Cover	E value	Per. Ident	Acc. Len	Accession
Alternaria arborescens mitovirus 1 isolate PMS8_29, complete genome	Alternaria arborescens mitovirus 1	917	917	99%	0.0	92.99%	2483	MN599397.1
Soybean leaf-associated mitovirus 2 isolate SaMitV2-1, partial sequence	Soybean leaf-associated mitovirus 2	865	865	100%	0.0	91.50%	2477	KT598239.1

Contig 12990, length 669 bp

Type of analysis: NCBI blastn, optimize for highly similar sequences (megablast)

Description	Scientific Name	Max Score	Total Score	Query Cover	E value	Per. Ident	Acc. Len	Accession
Alternaria arborescens mitovirus 1 isolate PMS8_29, complete genome	Alternaria arborescens mitovirus 1	1053	1053	100%	0.0	95.07%	2483	MN599397.1
Alternaria arborescens mitovirus 1 genomic RNA, complete genome	Alternaria arborescens mitovirus 1	970	970	100%	0.0	92.83%	2506	LC145036.1
Soybean leaf-associated mitovirus 2 isolate SaMitV2-1, partial sequence	Soybean leaf-associated mitovirus 2	568	568	79%	5e-157	86.17%	2477	KT598239.1

Contig 12723, length 681 bp

Type of analysis: NCBI blastn, optimize for highly similar sequences (megablast)

Description	Scientific Name	Max Score	Total Score	Query Cover	E value	Per. Ident	Acc. Len	Accession

Description	Scientific Name	Max Score	Total Score	Query Cover	E value	Per. Ident	Acc. Len	Accession
Alternaria arborescens mitovirus 1 isolate PMS8_29, complete genome	Alternaria arborescens mitovirus 1	1075	1075	100%	0.0	95.15%	2483	MN599397.1
Alternaria arborescens mitovirus 1 genomic RNA, complete genome	Alternaria arborescens mitovirus 1	1037	1037	100%	0.0	94.13%	2506	LC145036.1
Soybean leaf-associated mitovirus 2 isolate SaMitV2-1, partial sequence	Soybean leaf-associated mitovirus 2	804	804	100%	0.0	88.03%	2477	KT598239.1

Contig 5269, length 1432 bp

Type of analysis: NCBI blastn, optimize for highly similar sequences (megablast)

Description	Scientific Name	Max Score	Total Score	Query Cover	E value	Per. Ident	Acc. Len	Accession
Alternaria arborescens mitovirus 1 genomic RNA, complete genome	Alternaria arborescens mitovirus 1	2274	2274	100%	0.0	95.32%	2506	LC145036.1
Alternaria arborescens mitovirus 1 isolate PMS8_29, complete genome	Alternaria arborescens mitovirus 1	2228	2228	99%	0.0	94.82%	2483	MN599397.1
Soybean leaf-associated mitovirus 2 isolate SaMitV2-1, partial sequence	Soybean leaf-associated mitovirus 2	1773	1773	99%	0.0	89.05%	2477	KT598239.1

Contig 3627, length 1801 bp

Type of analysis: NCBI blastn, optimize for highly similar sequences (megablast)

Description	Scientific Name	Max Score	Total Score	Query Cover	E value	Per. Ident	Acc. Len	Accession
Alternaria arborescens mitovirus 1 genomic RNA, complete genome	Alternaria arborescens mitovirus 1	2846	2846	99%	0.0	95.22%	2506	LC145036.1
Alternaria arborescens mitovirus 1 isolate PMS8_29, complete genome	Alternaria arborescens mitovirus 1	2798	2798	99%	0.0	94.77%	2483	MN599397.1
Soybean leaf-associated mitovirus 2 isolate SaMitV2-1, partial sequence	Soybean leaf-associated mitovirus 2	2444	2444	99%	0.0	91.16%	2477	KT598239.1

Contig 2236, length 2269 bp

Type of analysis: NCBI blastn, optimize for highly similar sequences (megablast)

Description	Scientific Name	Max Score	Total Score	Query Cover	E value	Per. Ident	Acc. Len	Accession
Alternaria arborescens mitovirus 1 isolate PMS8_29, complete genome	Alternaria arborescens mitovirus 1	3602	3602	99%	0.0	95.37%	2483	MN599397.1
Alternaria arborescens mitovirus 1 genomic RNA, complete genome	Alternaria arborescens mitovirus 1	3576	3576	100%	0.0	95.11%	2506	LC145036.1
Soybean leaf-associated mitovirus 2 isolate SaMitV2-1, partial sequence	Soybean leaf-associated mitovirus 2	2706	2706	93%	0.0	89.66%	2477	KT598239.1

Contig 2017, length 2377 bp

Type of analysis: NCBI blastn, optimize for highly similar sequences (megablast)

Description	Scientific Name	Max Score	Total Score	Query Cover	E value	Per. Ident	Acc. Len	Accession
Plasmopara viticola lesion associated mitovirus 7 isolate COPhil RNA-dependent RNA polymerase gene, complete cds	Plasmopara viticola lesion associated mitovirus 7	3834	3834	100%	0.0	95.79%	2377	MT822730.1
Plasmopara viticola lesion associated mitovirus 7 isolate DMG-D_DN27174 RNA-dependent RNA polymerase gene, complete cds	Plasmopara viticola lesion associated mitovirus 7	3735	3735	100%	0.0	95.04%	2394	MN539769.1
Sanya mitovirus 1 isolate QWXCSY216 genomic sequence	Sanya mitovirus 1	2488	2488	87%	0.0	88.33%	2079	MZ209933.1

Grapevine associated narnavirus-1

Contig 20796, length 402 bp

Type of analysis: NCBI blastn, optimize for highly similar sequences (megablast)

Description	Scientific Name	Max Score	Total Score	Query Cover	E value	Per. Ident	Acc. Len	Accession
Botrytis cinerea mitovirus 9 isolate BCS1_DN2958 RNA-dependent RNA polymerase gene, complete cds	Botrytis cinerea mitovirus 9	693	693	100%	0.0	97.76%	2720	MT089704.1
Grapevine associated narnavirus-1 RdRp gene for RNA dependent RNA polymerase, strain Ctg157, isolate HAZ3-5	Grapevine associated narnavirus-1	654	654	100%	0.0	96.02%	2735	LN827948.1
Grapevine associated narnavirus-1 RdRp gene for RNA dependent RNA polymerase, strain Ctg157, isolate HAZ1-4	Grapevine associated narnavirus-1	590	590	99%	6e-164	93.27%	2733	LN827943.1

Contig 20533, length 450 bp

Type of analysis: NCBI blastn, optimize for highly similar sequences (megablast)

Description	Scientific Name	Max Score	Total Score	Query Cover	E value	Per. Ident	Acc. Len	Accession
Grapevine associated narnavirus-1 RdRp gene for RNA dependent RNA polymerase, strain Ctg157, isolate HAZ3-5	Grapevine associated narnavirus-1	704	704	100%	0.0	94.89%	2735	LN827948.1
Botrytis cinerea mitovirus 9 isolate BCS1_DN2958 RNA-dependent RNA polymerase gene, complete cds	Botrytis cinerea mitovirus 9	682	682	100%	0.0	94.00%	2720	MT089704.1
Grapevine associated narnavirus-1 RdRp gene for RNA dependent RNA polymerase, strain Ctg157, isolate HAZ1-4	Grapevine associated narnavirus-1	627	627	100%	5e-175	91.80%	2733	LN827943.1
Erysiphe necator associated mitovirus 31 isolate PMS8_143, complete genome	Erysiphe necator associated mitovirus 31	566	566	100%	1e-156	89.33%	2701	MN611677.1

Contig 12590 length 689

Type of analysis: NCBI blastn, optimize for highly similar sequences (megablast)

Description	Scientific Name	Max Score	Total Score	Query Cover	E value	Per. Ident	Acc. Len	Accession
Botrytis cinerea mitovirus 9 isolate BCS1_DN2958 RNA-dependent RNA polymerase gene, complete cds	Botrytis cinerea mitovirus 9	1223	1223	100%	0.0	98.69%	2720	MT089704.1
Grapevine associated narnavirus-1 RdRp gene for RNA dependent RNA polymerase, strain Ctg157, isolate HAZ3-5	Grapevine associated narnavirus-1	1011	1011	96%	0.0	94.13%	2735	LN827948.1
Grapevine associated narnavirus-1 RdRp gene for RNA dependent RNA polymerase, strain Ctg157, isolate HAZ1-4	Grapevine associated narnavirus-1	854	854	99%	0.0	89.16%	2733	LN827943.1
Erysiphe necator associated mitovirus 31 isolate PMS8_143, complete genome	Erysiphe necator associated mitovirus 31	712	712	94%	0.0	86.77%	2701	MN611677.1

Contig 12348, length 701 bp

Type of analysis: NCBI blastn, optimize for highly similar sequences (megablast)

Description	Scientific Name	Max Score	Total Score	Query Cover	E value	Per. Ident	Acc. Len	Accession
Grapevine associated narnavirus-1 RdRp gene for RNA dependent RNA polymerase, strain Ctg157, isolate HAZ3-5	Grapevine associated narnavirus-1	1212	1212	100%	0.0	97.86%	2735	LN827948.1
Botrytis cinerea mitovirus 9 isolate BCS1_DN2958 RNA-dependent RNA polymerase gene, complete cds	Botrytis cinerea mitovirus 9	1201	1201	100%	0.0	97.57%	2720	MT089704.1
Grapevine associated narnavirus-1 RdRp gene for RNA dependent RNA polymerase, strain Ctg157, isolate HAZ1-4	Grapevine associated narnavirus-1	1042	1042	99%	0.0	93.56%	2733	LN827943.1
Erysiphe necator associated mitovirus 31 isolate PMS8_143, complete genome	Erysiphe necator associated mitovirus 31	935	935	98%	0.0	91.09%	2701	MN611677.1

Contig 12349, length 701 bp

Type of analysis: NCBI blastn, optimize for highly similar sequences (megablast)

Description	Scientific Name	Max Score	Total Score	Query Cover	E value	Per. Ident	Acc. Len	Accession
Botrytis cinerea mitovirus 9 isolate BCS1_DN2958 RNA-dependent RNA polymerase gene, complete cds	Botrytis cinerea mitovirus 9	1195	1195	100%	0.0	97.43%	2720	MT089704.1
Grapevine associated narnavirus-1 RdRp gene for RNA dependent RNA polymerase, strain Ctg157, isolate HAZ3-5	Grapevine associated narnavirus-1	1162	1162	100%	0.0	96.58%	2735	LN827948.1
Grapevine associated narnavirus-1 RdRp gene for RNA dependent RNA polymerase, strain Ctg157, isolate HAZ1-4	Grapevine associated narnavirus-1	1031	1031	99%	0.0	93.28%	2733	LN827943.1
Erysiphe necator associated mitovirus 31 isolate PMS8_143, complete genome	Erysiphe necator associated mitovirus 31	918	918	98%	0.0	90.67%	2701	MN611677.1

Contig 10213, length 837 bp

Type of analysis: NCBI blastn, optimize for highly similar sequences (megablast)

Description	Scientific Name	Max Score	Total Score	Query Cover	E value	Per. Ident	Acc. Len	Accession
Botrytis cinerea mitovirus 9 isolate BCS1_DN2958 RNA-dependent RNA polymerase gene, complete cds	Botrytis cinerea mitovirus 9	1452	1452	100%	0.0	97.97%	2720	MT089704.1
Grapevine associated narnavirus-1 RdRp gene for RNA dependent RNA polymerase, strain Ctg157, isolate HAZ3-5	Grapevine associated narnavirus-1	1391	1391	100%	0.0	96.66%	2735	LN827948.1
Grapevine associated narnavirus-1 RdRp gene for RNA dependent RNA polymerase, strain Ctg157, isolate HAZ1-4	Grapevine associated narnavirus-1	1192	1192	100%	0.0	92.39%	2733	LN827943.1
Erysiphe necator associated mitovirus 31 isolate PMS8_143, complete genome	Erysiphe necator associated mitovirus 31	992	992	99%	0.0	88.26%	2701	MN611677.1

Contig 10204, length 838 bp

Type of analysis: NCBI blastn, optimize for highly similar sequences (megablast)

Description	Scientific Name	Max Score	Total Score	Query Cover	E value	Per. Ident	Acc. Len	Accession
Botrytis cinerea mitovirus 9 isolate BCS1_DN2958 RNA-dependent RNA polymerase gene, complete cds	Botrytis cinerea mitovirus 9	1459	1459	100%	0.0	98.09%	2720	MT089704.1
Grapevine associated narnavirus-1 RdRp gene for RNA dependent RNA polymerase, strain Ctg157, isolate HAZ3-5	Grapevine associated narnavirus-1	1386	1386	100%	0.0	96.54%	2735	LN827948.1
Grapevine associated narnavirus-1 RdRp gene for RNA dependent RNA polymerase, strain Ctg157, isolate HAZ1-4	Grapevine associated narnavirus-1	1210	1210	100%	0.0	92.74%	2733	LN827943.1
Erysiphe necator associated mitovirus 31 isolate PMS8_143, complete genome	Erysiphe necator associated mitovirus 31	998	998	99%	0.0	88.40%	2701	MN611677.1

Sclerotinia sclerotiorum mitovirus 2

Contig 9935, length 860 bp

Type of analysis: NCBI blastn, optimize for highly similar sequences (megablast)

Description	Scientific Name	Max Score	Total Score	Query Cover	E value	Per. Ident	Acc. Len	Accession
Plasmopara viticola lesion associated mitovirus 39 isolate DMG-E_DN25361 RNA-dependent RNA polymerase gene, complete cds	Plasmopara viticola lesion associated mitovirus 39	1463	1463	99%	0.0	97.65%	2339	MN539800.1
Erysiphe necator associated mitovirus 25 isolate PMS1_135, complete genome	Erysiphe necator associated mitovirus 25	1452	1452	98%	0.0	97.53%	2353	MN611671.1
Erysiphe necator associated mitovirus 14 isolate PMS12_11, complete genome	Erysiphe necator associated mitovirus 14	813	813	100%	0.0	83.80%	2385	MN611660.1
Plasmopara viticola lesion associated mitovirus 38 isolate DMS8_DN22923 RNA-dependent RNA polymerase gene, complete cds	Plasmopara viticola lesion associated mitovirus 38	795	795	99%	0.0	83.45%	2348	MN539799.1

Sclerotinia sclerotiorum mitovirus 3

Contig 12938, length 672 bp

Type of analysis: NCBI blastn, optimize for highly similar sequences (megablast)

Description	Scientific Name	Max Score	Total Score	Query Cover	E value	Per. Ident	Acc. Len	Accession
Sclerotinia sclerotiorum mitovirus 3 RNA-dependent RNA polymerase gene, complete cds	Sclerotinia sclerotiorum mitovirus 3	965	965	100%	0.0	92.57%	2588	JX401537.1
Erysiphe necator associated mitovirus 27 isolate PMS5_DN58702, complete genome	Erysiphe necator associated mitovirus 27	959	959	100%	0.0	92.42%	2530	MN611673.1
Sclerotinia sclerotiorum mitovirus 3 isolate BCI2_DN28, complete genome	Sclerotinia sclerotiorum mitovirus 3	948	948	100%	0.0	92.12%	2974	MN954876.1
Sclerotinia sclerotiorum mitovirus 3 RdRp gene for RNA	Sclerotinia sclerotiorum	937	937	100%	0.0	91.83%	2617	LN827949.1

Description	Scientific Name	Max Score	Total Score	Query Cover	E value	Per. Ident	Acc. Len	Accession
dependent RNA polymerase, isolate HAZ3-6	mitovirus 3							
Sclerotinia nivalis mitovirus 2 isolate SsSn-1.m2, complete genome	Sclerotinia nivalis mitovirus 2	898	898	100%	0.0	90.80%	2583	KT365896.1
Sclerotinia sclerotiorum mitovirus 3-WX, partial genome	Sclerotinia sclerotiorum mitovirus 3-WX	876	876	100%	0.0	90.21%	2581	MT646425.1

Contig 1764, length 2525 bp

Type of analysis: NCBI blastn, optimize for highly similar sequences (megablast)

Description	Scientific Name	Max Score	Total Score	Query Cover	E value	Per. Ident	Acc. Len	Accession
Sclerotinia sclerotiorum mitovirus 3 RNA-dependent RNA polymerase gene, complete cds	Sclerotinia sclerotiorum mitovirus 3	4109	4109	100%	0.0	96.05%	2588	JX401537.1
Sclerotinia sclerotiorum mitovirus 3 RdRp gene for RNA dependent RNA polymerase, isolate HAZ3-6	Sclerotinia sclerotiorum mitovirus 3	3642	3642	99%	0.0	92.74%	2617	LN827949.1
Sclerotinia sclerotiorum mitovirus 3-WX, partial genome	Sclerotinia sclerotiorum mitovirus 3-WX	3618	3618	100%	0.0	92.54%	2581	MT646425.1
Erysiphe necator associated mitovirus 27 isolate PMS5_DN58702, complete genome	Erysiphe necator associated mitovirus 27	3596	3596	99%	0.0	92.42%	2530	MN611673.1
Sclerotinia nivalis mitovirus 2 isolate SsSn-1.m2, complete genome	Sclerotinia nivalis mitovirus 2	3518	3518	100%	0.0	91.87%	2583	KT365896.1

Sclerotinia sclerotiorum negative-stranded virus 1

Contig 10393, length 825 bp

Type of analysis: NCBI blastn, optimize for highly similar sequences (megablast)

Description	Scientific Name	Max Score	Total Score	Query Cover	E value	Per. Ident	Acc. Len	Accession
Botrytis cinerea negative-stranded RNA virus 3 isolate BCS11_19, complete genome	Botrytis cinerea negative-stranded RNA virus 3	1338	1338	99%	0.0	95.99%	9875	MN617150.1
Sclerotinia sclerotiorum negative-stranded RNA virus 9, partial genome	Sclerotinia sclerotiorum negative-stranded RNA virus 9	1122	1122	99%	0.0	91.25%	9624	MT646386.1

Sclerotinia sclerotiorum negative-stranded virus 3

Contig 7324, length 1124 bp

Type of analysis: NCBI blastn, optimize for highly similar sequences (megablast)

Description	Scientific Name	Max Score	Total Score	Query Cover	E value	Per. Ident	Acc. Len	Accession
Botrytis cinerea negative-stranded RNA virus 3 isolate BCS11_19, complete genome	Botrytis cinerea negative-stranded RNA virus 3	1803	1803	99%	0.0	95.64%	9875	MN617150.1
Sclerotinia sclerotiorum negative-stranded RNA virus 9, partial genome	Sclerotinia sclerotiorum negative-stranded RNA virus 9	1583	1583	99%	0.0	92.15%	9624	MT646386.1

Contig 4664, length 1551 bp

Type of analysis: NCBI blastn, optimize for highly similar sequences (megablast)

Description	Scientific Name	Max Score	Total Score	Query Cover	E value	Per. Ident	Acc. Len	Accession
Vitis vinifera contig VV78X039104.5, whole genome shotgun sequence	Vitis vinifera	2089	2089	79%	0.0	96.85%	22513	AM474284.2
Vitis rotundifolia cultivar Noble chromosome 18	Vitis rotundifolia	1022	1821	72%	0.0	95.08%	30244861	CP092943.1
PREDICTED: Vitis vinifera uncharacterized LOC100267991 (LOC100267991), mRNA	Vitis vinifera	981	981	38%	0.0	96.62%	682	XM_002263167.4
PREDICTED: Vitis riparia uncharacterized LOC117927172 (LOC117927172), transcript variant X1, mRNA	Vitis riparia	808	879	33%	0.0	97.29%	533	XM_034846603.1
PREDICTED: Vitis riparia uncharacterized LOC117927172 (LOC117927172), transcript variant X2, mRNA	Vitis riparia	802	877	33%	0.0	97.27%	555	XM_034846604.1
Botrytis cinerea negative-stranded RNA virus 3 isolate BCS11_19, complete genome	Botrytis cinerea negative-stranded RNA virus 3	523	523	20%	3e-143	96.52%	9875	MN617150.1
Sclerotinia sclerotiorum negative-stranded RNA virus 9, partial genome	Sclerotinia sclerotiorum negative-stranded RNA virus 9	412	412	20%	6e-110	90.19%	9624	MT646386.1

Contig 4660, length 1552 bp

Type of analysis: NCBI blastn, optimize for highly similar sequences (megablast)

Description	Scientific Name	Max Score	Total Score	Query Cover	E value	Per. Ident	Acc. Len	Accession
Vitis vinifera contig VV78X039104.5, whole genome shotgun sequence	Vitis vinifera	2172	2172	79%	0.0	98.06%	22513	AM474284.2

Description	Scientific Name	Max Score	Total Score	Query Cover	E value	Per. Ident	Acc. Len	Accession
Vitis rotundifolia cultivar Noble chromosome 18	Vitis rotundifolia	1103	1902	72%	0.0	97.24%	30244861	CP092943.1
PREDICTED: Vitis vinifera uncharacterized LOC100267991 (LOC100267991), mRNA	Vitis vinifera	1053	1053	38%	0.0	98.82%	682	XM_002263167.4
PREDICTED: Vitis riparia uncharacterized LOC117927172 (LOC117927172), transcript variant X1, mRNA	Vitis riparia	824	896	33%	0.0	97.91%	533	XM_034846603.1
PREDICTED: Vitis riparia uncharacterized LOC117927172 (LOC117927172), transcript variant X2, mRNA	Vitis riparia	819	894	33%	0.0	97.90%	555	XM_034846604.1
Botrytis cinerea negative-stranded RNA virus 3 isolate BCS11_19, complete genome	Botrytis cinerea negative-stranded RNA virus 3	523	523	20%	3e-143	96.52%	9875	MN617150.1
Sclerotinia sclerotiorum negative-stranded RNA virus 9, partial genome	Sclerotinia sclerotiorum negative-stranded RNA virus 9	412	412	20%	6e-110	90.19%	9624	MT646386.1

Contig 4639, length 1558 bp

Type of analysis: NCBI blastn, optimize for highly similar sequences (megablast)

Description	Scientific Name	Max Score	Total Score	Query Cover	E value	Per. Ident	Acc. Len	Accession
Vitis vinifera contig VV78X039104.5, whole genome shotgun sequence	Vitis vinifera	2119	2119	79%	0.0	97.11%	22513	AM474284.2
Vitis rotundifolia cultivar Noble chromosome 18	Vitis rotundifolia	1046	1845	73%	0.0	95.19%	30244861	CP092943.1
PREDICTED: Vitis vinifera uncharacterized LOC100267991 (LOC100267991), mRNA	Vitis vinifera	1003	1003	37%	0.0	97.30%	682	XM_002263167.4
PREDICTED: Vitis riparia uncharacterized LOC117927172	Vitis riparia	808	879	33%	0.0	97.29%	533	XM_034846

Description	Scientific Name	Max Score	Total Score	Query Cover	E value	Per. Ident	Acc. Len	Accession
(LOC117927172), transcript variant X1, mRNA								603.1
PREDICTED: Vitis riparia uncharacterized LOC117927172 (LOC117927172), transcript variant X2, mRNA	Vitis riparia	802	877	33%	0.0	97.27%	555	XM_034846 604.1
Botrytis cinerea negative-stranded RNA virus 3 isolate BCS11_19, complete genome	Botrytis cinerea negative-stranded RNA virus 3	523	523	20%	3e-143	96.52%	9875	MN617150.1
Sclerotinia sclerotiorum negative-stranded RNA virus 9, partial genome	Sclerotinia sclerotiorum negative-stranded RNA virus 9	412	412	20%	6e-110	90.19%	9624	MT646386.1

Contig 2441, length 2171 bp

Type of analysis: NCBI blastn, optimize for highly similar sequences (megablast)

Description	Scientific Name	Max Score	Total Score	Query Cover	E value	Per. Ident	Acc. Len	Accession
Botrytis cinerea negative-stranded RNA virus 3 isolate BCS11_19, complete genome	Botrytis cinerea negative-stranded RNA virus 3	3358	3358	100%	0.0	94.57%	9875	MN617150.1
Sclerotinia sclerotiorum negative-stranded RNA virus 9, partial genome	Sclerotinia sclerotiorum negative-stranded RNA virus 9	2946	2946	100%	0.0	91.20%	9624	MT646386.1

Contig 1113, length 3037 bp

Type of analysis: NCBI blastn, optimize for highly similar sequences (megablast)

Description	Scientific Name	Max Score	Total Score	Query Cover	E value	Per. Ident	Acc. Len	Accession
Botrytis cinerea negative-stranded RNA virus 3 isolate	Botrytis cinerea negative-	4756	4756	99%	0.0	94.96%	9875	MN617150.1

Description	Scientific Name	Max Score	Total Score	Query Cover	E value	Per. Ident	Acc. Len	Accession
BCS11_19, complete genome	stranded RNA virus 3							
Sclerotinia sclerotiorum negative-stranded RNA virus 9, partial genome	Sclerotinia sclerotiorum negative-stranded RNA virus 9	4224	4224	100%	0.0	91.77%	9624	MT646386.1
