# Supplementary material for: The First Virome of a Russian Vineyard
Source: Plants (Basel). 2023 Sep 18;12(18):3292. doi: 10.3390/plants12183292 (PMC10534617; doi:10.3390/plants12183292)
Supplement: Supplementary file 1 [file plants-12-03292-s001.zip › Supplementary Figures Vinogradova et al.pdf]

The first virome of Russian vineyard

Supplementary materials

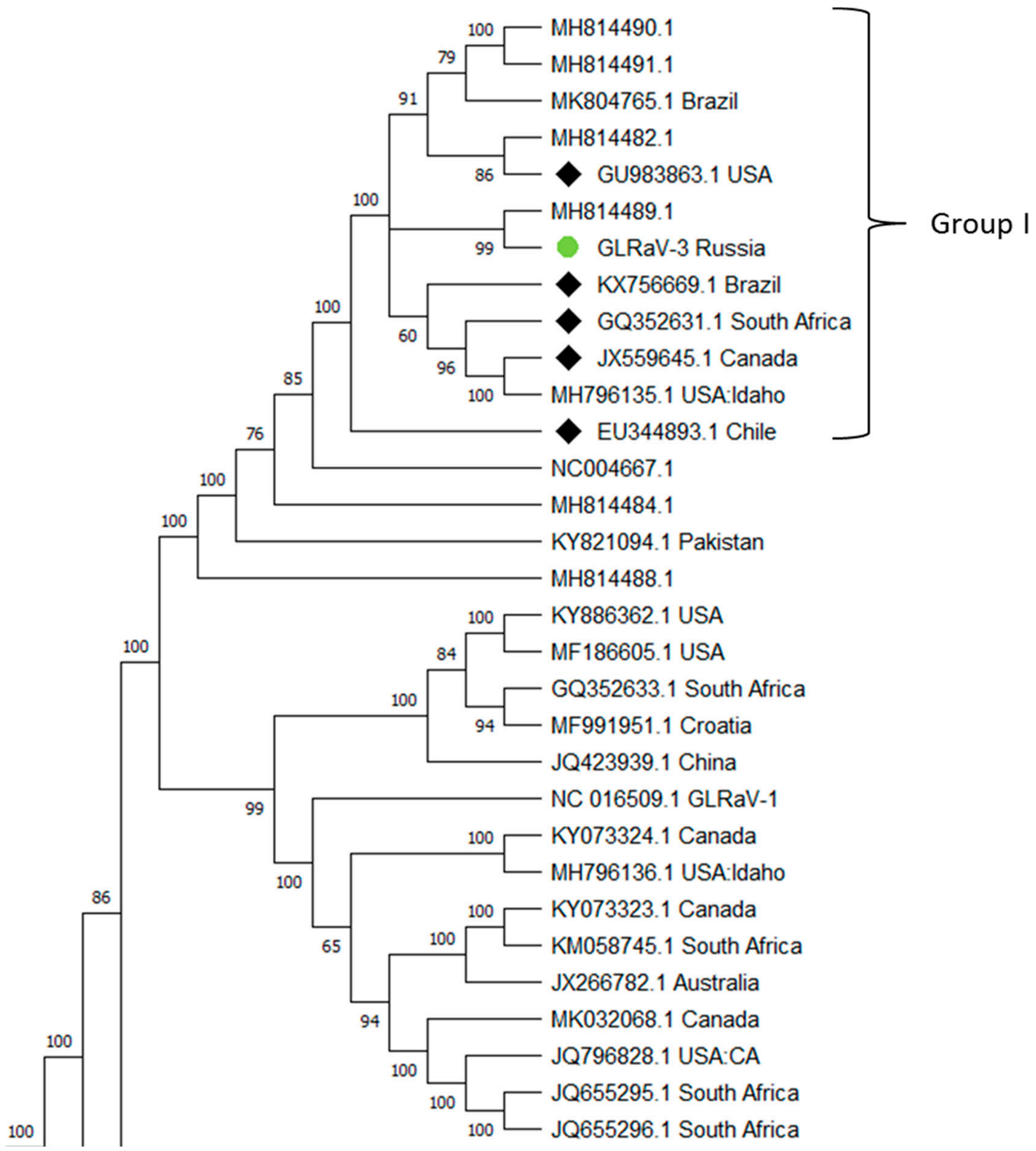

Supplementary Figure S1. Part of the phylogenetic tree showing the distribution of nucleotide sequences of complete genome in Russian isolates of grapevine leafroll-associated virus 3 ( ) compared to isolates from the GenBank and representative sequences (◆). Geographic origin is indicated for each Russian isolate (in brackets). The tree is rooted, but the outgroups have been removed for ease of presentation. Bootstrap values >60% (1,000 bootstrap replicates) are shown. The tree is rooted, but the outgroups have been removed for ease of presentation.

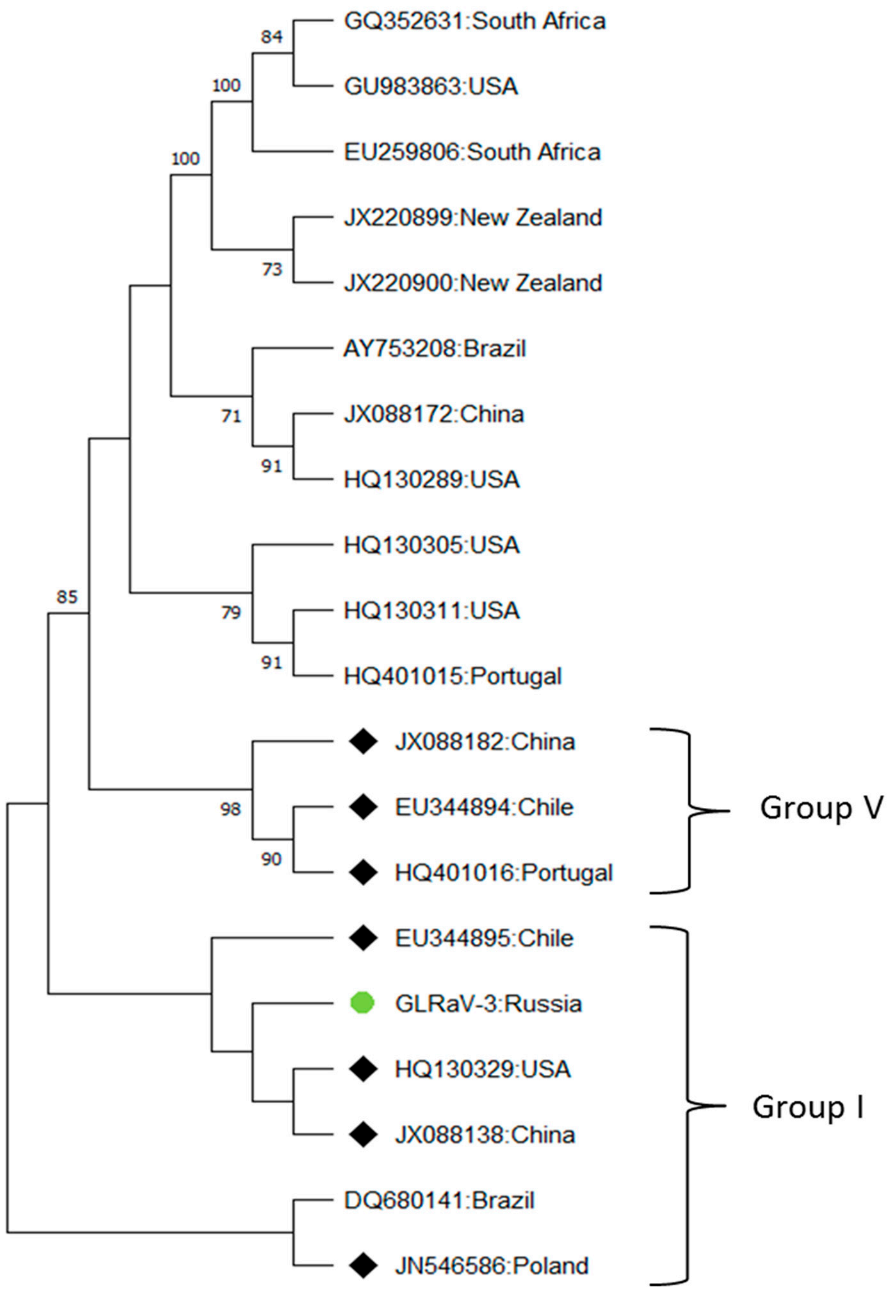

Supplementary Figure S2. Phylogenetic tree showing the distribution of nucleotide sequences of coat protein genes in Russian isolates of grapevine leafroll-associated virus 3 ( ) compared to isolates from the GenBank and representative sequences (◆). Geographic origin is indicated for each Russian isolate (in brackets). Bootstrap values >60% (1,000 bootstrap replicates) are shown.

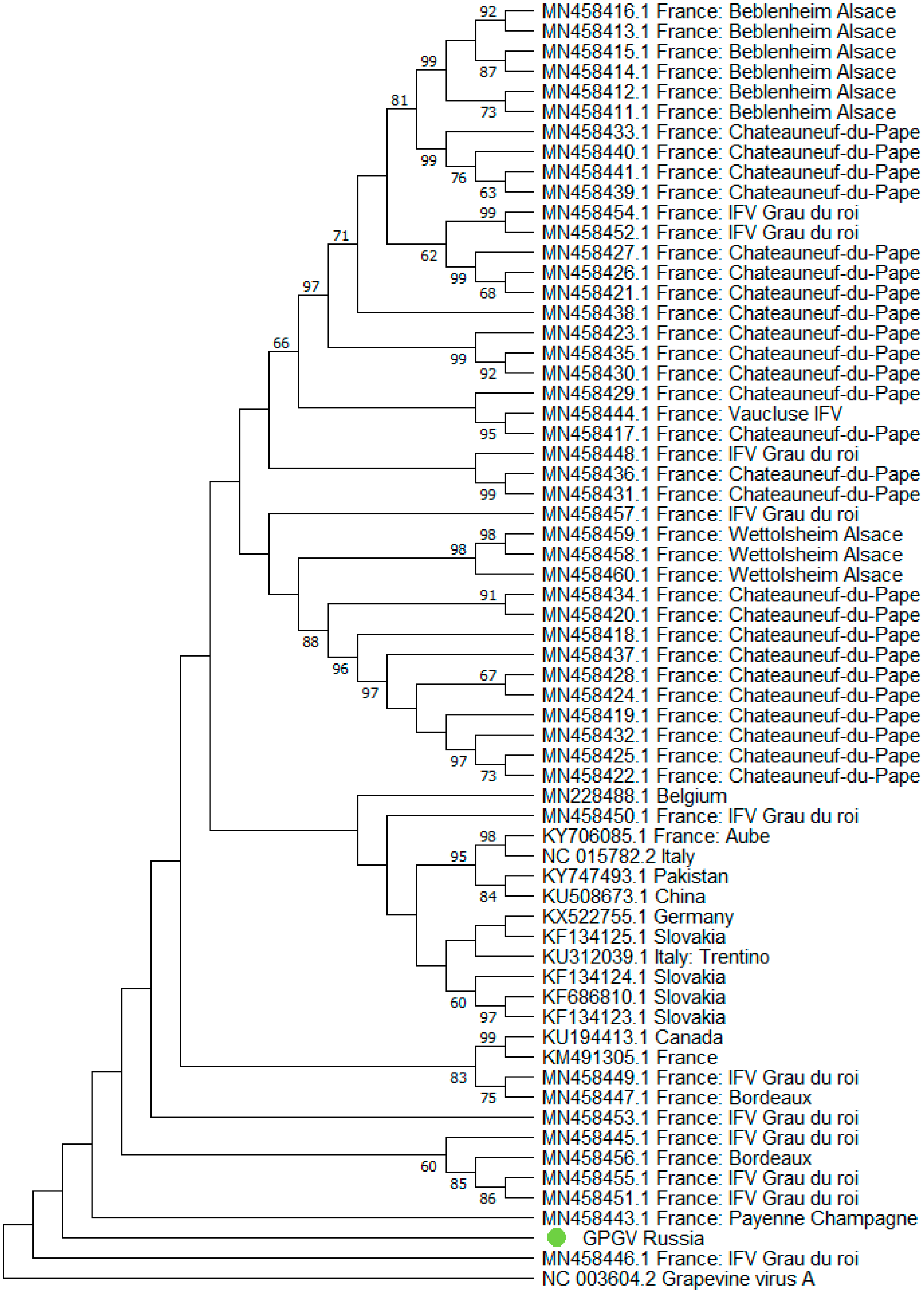

Supplementary Figure S3. Phylogenetic tree showing the distribution of nucleotide sequences of complete genome in Russian isolates of grapevine Pinot gris virus (●) compared to isolates from the GenBank and representative sequences (◆). Geographic origin is indicated for each Russian isolate (in brackets). Bootstrap values >60% (1,000 bootstrap replicates) are shown. The tree is rooted.

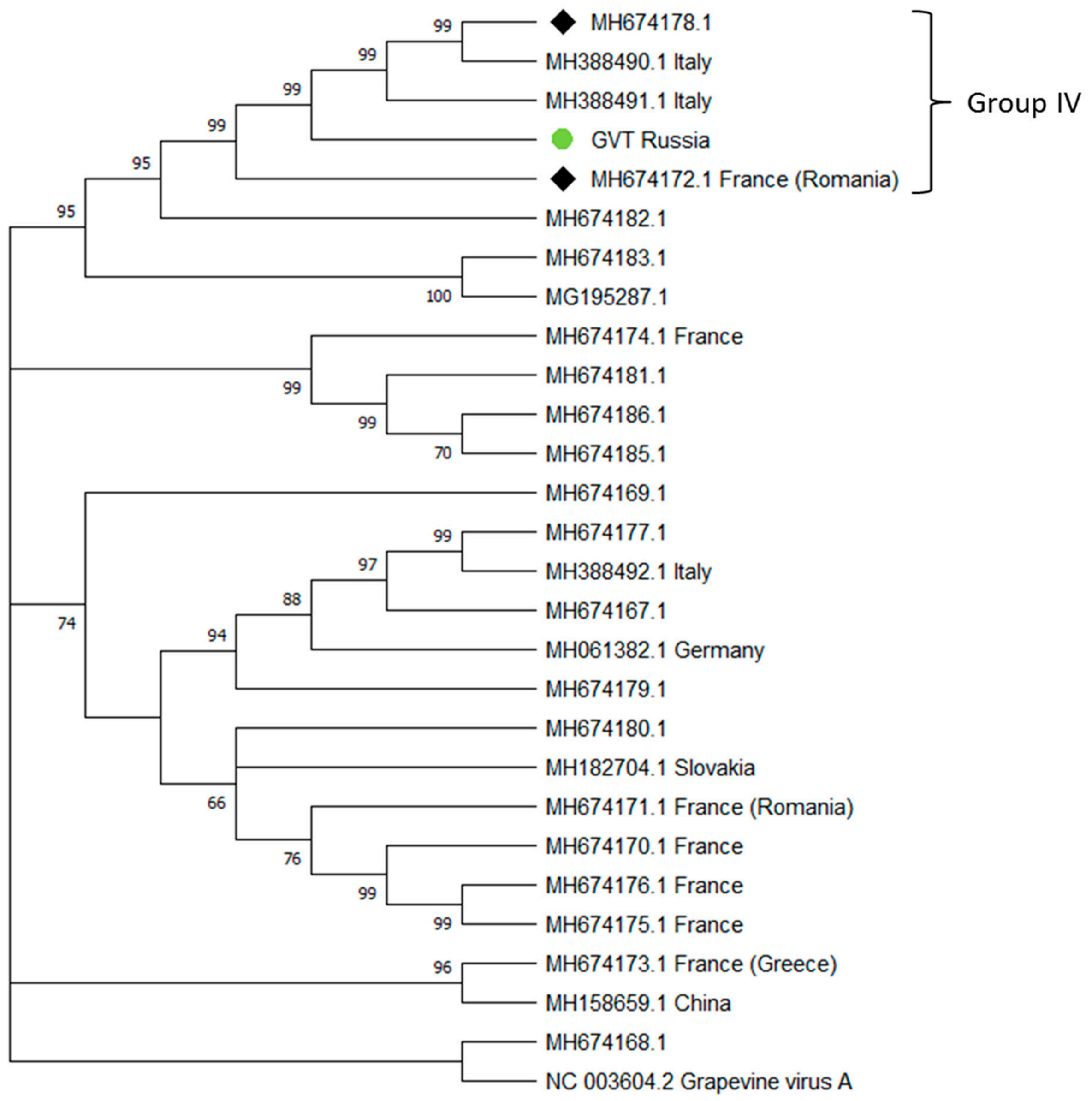

Supplementary Figure S4. Phylogenetic tree showing the distribution of nucleotide sequences of complete genome in Russian isolates of grapevine virus T (■) compared to isolates from the GenBank and representative sequences (◆). Geographic origin is indicated for each Russian isolate (in brackets). Bootstrap values >60% (1,000 bootstrap replicates) are shown. The tree is rooted.

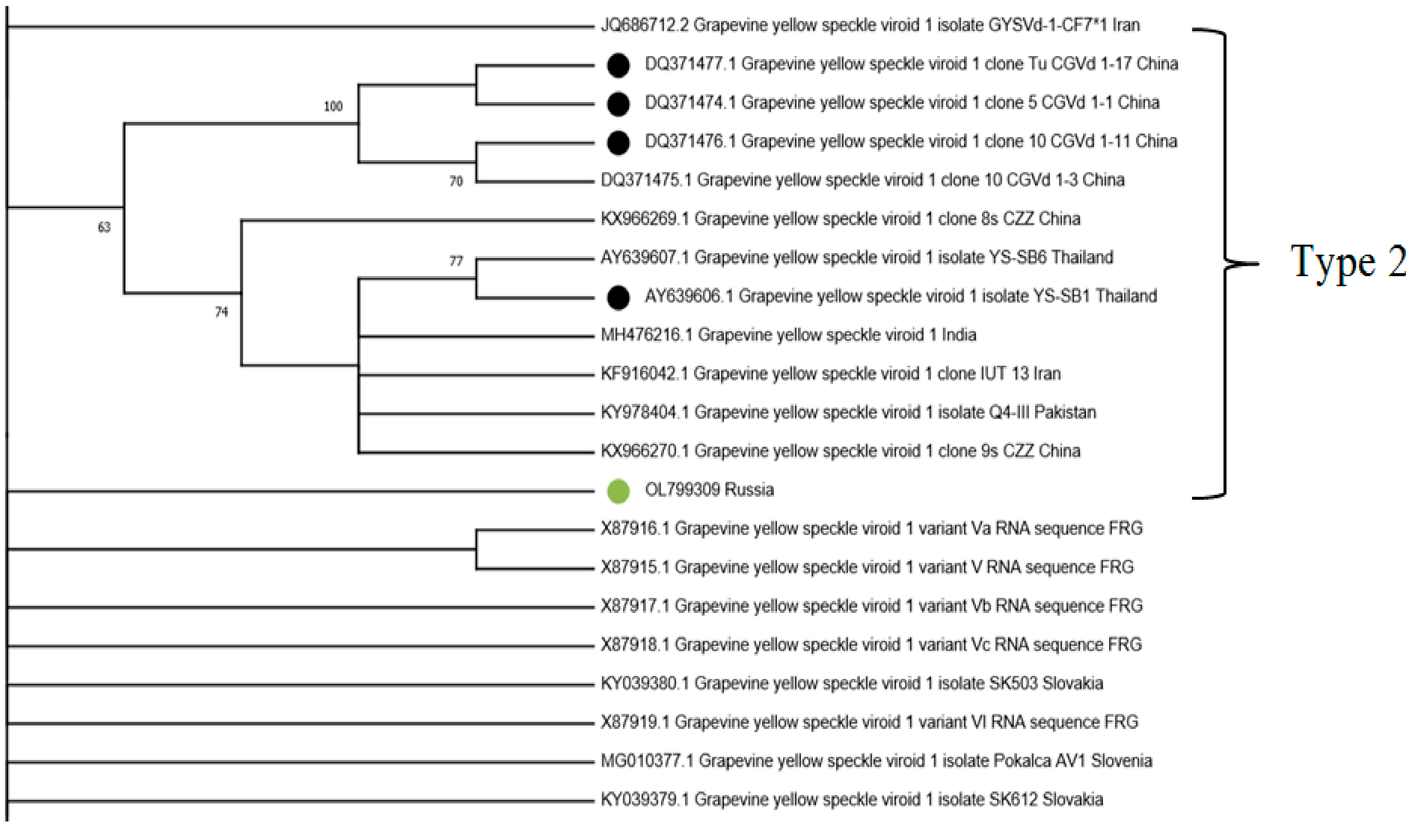

Supplementary Figure S5. Part of the phylogenetic tree showing the distribution of nucleotide sequences of complete genome in Russian isolates of grapevine yellow speckle viroid 1 ( ) compared to isolates from the GenBank and representative sequences ( ). Geographic origin is indicated for each Russian isolate (in brackets). Bootstrap values >60% (1,000 bootstrap replicates) are shown. The tree is rooted, but the outgroups have been removed for ease of presentation.

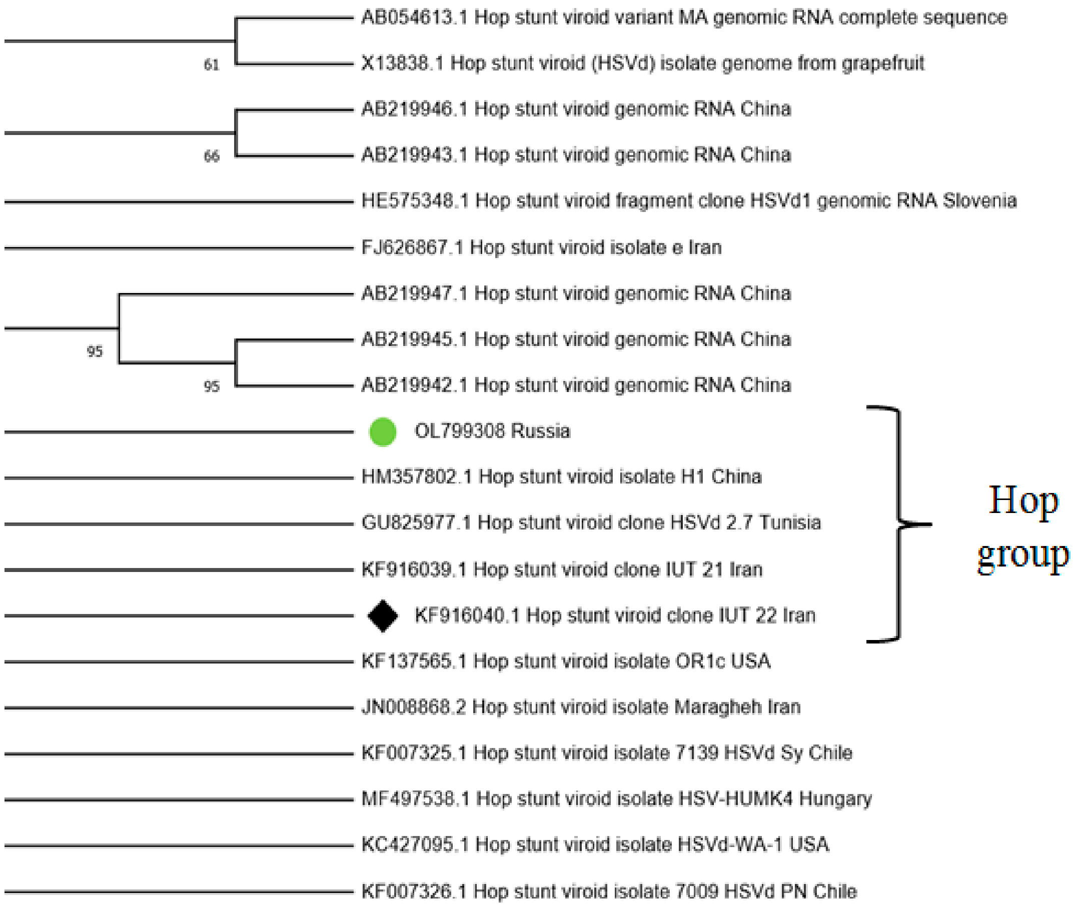

Supplementary Figure S6. Part of the phylogenetic tree showing the distribution of nucleotide sequences of complete genome in Russian isolates of hop stunt viroid (■) compared to isolates from the GenBank and representative sequences (◆). Geographic origin is indicated for each Russian isolate (in brackets). Bootstrap values >60% (1,000 bootstrap replicates) are shown. The tree is rooted, but the outgroups have been removed for ease of presentation.
