# Supplementary material for: The First Virome of a Russian Vineyard
Source: Plants (Basel). 2023 Sep 18;12(18):3292. doi: 10.3390/plants12183292 (PMC10534617; doi:10.3390/plants12183292)
Supplement: Supplementary file 1 [file plants-12-03292-s001.zip › Supplementary Validation of additional mycoviruses Vinogradova et al.pdf]

Supplementary materials

Validation of additional mycoviruses

Plasmopara viticola lesion associated ourmia-like virus 82 isolate 605

Type of analysis: NCBI blastn, optimize for highly similar sequences (megablast)

AATCCCTCAGAAATCTCCTCTTTGTTCCAATTCCCTCCGACATTTCTACCGAACGATTCGGTTGCGTGGCCGTTGGGAATAAAAGGACGACGACGATCATCCCAGCCGTACGGCACATTT
CGGTCAAACGCCAAACTGAACCTGGCAAGGTGATCATCATCGACCTCCACCCATCGCAACCGAGCCTCCTTCCAAGAGCCCAGCCTCTCAAGTGCAGCGGGTTCGCACCTAGTACAAA
CCCTCTTTTCAATTTTCTGCACTGTTTTGAAACTAAGCTCCCACACCGGAGACACAGCAGGGAAGCAAGACCGAACTGCCTGGCGCAGTTCGCCGCATCTCACTCTCTCTGGGAGATCC
CCGGACGCAGCAAGTCCATGGGACTTAAACCACCTCACAAGGGTTCTAGCCTTCCCCCTAAGGCTATCGGAAAATAGACAATCACCGTCATCGTCAGATAACACCGCA

Description	Scientific Name	Max Score	Total Score	Query Cover	E value	Per. Ident	Acc. Len	Accession
Plasmopara viticola lesion associated ourmia-like virus 82 isolate DMS4_33408 RNA dependent RNA polymerase gene, partial cds	Plasmopara viticola lesion associated ourmia-like virus 82	616	616	100%	1e-171	90.56%	3324	MN532669.1

Sclerotinia sclerotiorum mitovirus 4 isolate 593

Type of analysis: NCBI blastn, optimize for highly similar sequences (megablast)

CGGACTTGGACGGTACGTAGTGTTAACTGACACTATCTAAATTCAAGATTCTCATGAATAATAAAATTAGAAATATAATCATAATAAGGTTATTAAGACTATGTTTCAACTTTAATAATA
ATACATGAGAAATTACTCGGTTTTTGGAAACCTTCGATGAAATGAGACAAAAGTCTGGTCTTAAATATACTATCAAGTATTATAAAGCTGTAAAGCTTCATATTACTAGATATATATGTG

GACAACCTTTATTCTCAAACAAAGAAGGAGTTGCTTTAGATCATTCTGGTTGACCTAAGAAATTCTTATATCTTAAAAAGTTTATTAAAACAAATAAAGAATTAAGAATTCTATTAACTC
TTCTATCTTTTACAAGAACAGTTGTTCCTACAAAAGTAGAAGAATTAAAGATCAAACCAGATTATTCAACAATTGATTTACCATACAAAGGGAAAAGTTATACTATCCCTGCA

Description	Scientific Name	Max Score	Total Score	Query Cover	E value	Per. Ident	Acc. Len	Accession
Sclerotinia sclerotiorum mitovirus 4-A isolate SsMV4-A RNA-dependent RNA polymerase gene, complete cds	Sclerotinia sclerotiorum mitovirus 4-A	719	719	100%	0.0	94.08%	2737	MF444237.1
Sclerotinia sclerotiorum mitovirus 4 isolate BCS16_24, complete genome	Sclerotinia sclerotiorum mitovirus 4	713	713	100%	0.0	93.88%	2737	MN954877.1
Sclerotinia sclerotiorum mitovirus 4, complete sequence	Sclerotinia sclerotiorum mitovirus 4	712	712	99%	0.0	93.87%	2752	KT962974.1
Botrytis cinerea mitovirus 4 isolate BCS16_23, complete genome	Botrytis cinerea mitovirus 4	697	697	92%	0.0	95.43%	2709	MN954875.1
Sclerotinia sclerotiorum mitovirus 4 RNA-dependent RNA polymerase gene, complete cds	Sclerotinia sclerotiorum mitovirus 4	667	667	99%	0.0	92.18%	2744	JX401538.1
Botrytis cinerea mitovirus 4 RdRp gene for RNA dependent RNA polymerase, isolate HAZ3-4	Botrytis cinerea mitovirus 4	364	364	92%	5e-96	81.94%	2768	LN827947.1
Sclerotinia sclerotiorum mitovirus 36, partial genome	Sclerotinia sclerotiorum mitovirus 36	348	348	92%	5e-91	81.31%	2732	MT646380.1
Sclerotinia nivalis mitovirus 1 isolate SsSn-1.m1, complete genome	Sclerotinia nivalis mitovirus 1	327	327	97%	6e-85	79.74%	2720	KT365895.1

Description	Scientific Name	Max Score	Total Score	Query Cover	E value	Per. Ident	Acc. Len	Accession
Botrytis cinerea mitovirus 7 isolate BCI5_DN3722, complete genome	Botrytis cinerea mitovirus 7	285	285	94%	4e-72	78.40%	2705	MN617168.1

Sclerotinia sclerotiorum mitovirus 4 isolate 599

Type of analysis: NCBI blastn, optimize for highly similar sequences (megablast)

TCTATCTAAATTCAAGATTCTCATGAATAATAAAATTAGAAATATAATCATAATAAGGTTATTAAGACTATGTTTCAATTTTAATAATAATACATGAGAAATTACTCGGTTTTTGGAAACC
TTAGATGAGATGAGACAGAAGTCTGGTCTTAAATATACTATCAAATATTATAAAGCTGTAAAGCTTCATATTACAAGATATATATGTGGACAGCCTCTGTTATCAAACAAAGAAGGAGT
TGCTTTAGATCATTCTGGTTGACCAAAGAAATTCTTATATCTTAAAAAGTTTATAAAAACTAATAAAGAATTAAGAATTTTATTAACTCTTCTATCTTTTACAAGAACAGTTGTTCCTAGT
AAAAAGGAAGAGTTAAAGATCAAACCAGATTATTCAACAATTGATTTACCATACAAAGGAAAAGTATATACTATTCCTGCATGATT

Description	Scientific Name	Max Score	Total Score	Query Cover	E value	Per. Ident	Acc. Len	Accession
Sclerotinia sclerotiorum mitovirus 4 isolate BCS16_24, complete genome	Sclerotinia sclerotiorum mitovirus 4	693	693	99%	0.0	94.82%	2737	MN954877.1
Botrytis cinerea mitovirus 4 isolate BCS16_23, complete genome	Botrytis cinerea mitovirus 4	693	693	100%	0.0	94.64%	2709	MN954875.1
Sclerotinia sclerotiorum mitovirus 4 RNA-dependent RNA polymerase gene, complete cds	Sclerotinia sclerotiorum mitovirus 4	676	676	99%	0.0	94.14%	2744	JX401538.1
Sclerotinia sclerotiorum mitovirus 4, complete sequence	Sclerotinia sclerotiorum mitovirus 4	651	651	98%	0.0	93.23%	2752	KT962974.1

Description	Scientific Name	Max Score	Total Score	Query Cover	E value	Per. Ident	Acc. Len	Accession
Sclerotinia sclerotiorum mitovirus 4-A isolate SsMV4-A RNA-dependent RNA polymerase gene, complete cds	Sclerotinia sclerotiorum mitovirus 4-A	636	636	99%	8e-178	92.57%	2737	MF444237.1
Botrytis cinerea mitovirus 4 RdRp gene for RNA dependent RNA polymerase, isolate HAZ3-4	Botrytis cinerea mitovirus 4	326	326	98%	2e-84	80.18%	2768	LN827947.1
Sclerotinia sclerotiorum mitovirus 39, partial genome	Sclerotinia sclerotiorum mitovirus 39	313	313	94%	2e-80	80.24%	2614	MT646411.1
Sclerotinia nivalis mitovirus 1 isolate SsSn-1.m1, complete genome	Sclerotinia nivalis mitovirus 1	303	303	97%	1e-77	79.32%	2720	KT365895.1
Sclerotinia sclerotiorum mitovirus 36, partial genome	Sclerotinia sclerotiorum mitovirus 36	298	298	98%	5e-76	79.19%	2732	MT646380.1

Plasmopara viticola lesion associated mitovirus 39 isolate 594

Type of analysis: NCBI blastn, optimize for highly similar sequences (megablast)

TGGGACCGAATCTCTTTAACTCACAGGGAATATCTTCCTGTTATATCTAGTTTAGACAAGTCAACATCATTAAAGATGGAAAACTTGATAAACGTAAGAGAGTTAAAGTCCGGGTTGGA
CGCCTCTAAAACATTGGAATCTGACCCTATAAGGCCAGTTCCTAGTTTAAAAGGATTACAACCTATACGTCCTAATGAG

Description	Scientific Name	Max Score	Total Score	Query Cover	E value	Per. Ident	Acc. Len	Accession

[Description](#)	[Scientific Name](#)	Max Score	Total Score	Query Cover	E value	Per. Ident	Acc. Len	Accession
Erysiphe necator associated mitovirus 25 isolate PMS1_135, complete genome	Erysiphe necator associated mitovirus 25	333	333	100%	5e-87	96.97%	2353	MN611671.1
Plasmopara viticola lesion associated mitovirus 39 isolate DMG-E_DN25361 RNA-dependent RNA polymerase gene, complete cds	Plasmopara viticola lesion associated mitovirus 39	327	327	100%	2e-85	96.46%	2339	MN539800.1
Erysiphe necator associated mitovirus 14 isolate PMS12_11, complete genome	Erysiphe necator associated mitovirus 14	239	239	100%	1e-58	88.38%	2385	MN611660.1
Plasmopara viticola lesion associated mitovirus 38 isolate DMS8_DN22923 RNA-dependent RNA polymerase gene, complete cds	Plasmopara viticola lesion associated mitovirus 38	231	231	99%	2e-56	87.88%	2348	MN539799.1

Erysiphe necator mitovirus 1 isolate 603L

Type of analysis: NCBI blastn, optimize for highly similar sequences (megablast)

TGAGAACTTATTCAAGAACCAATTAACTCAATTGGATATTGAGCAATCTTCCAAATGGAATGCACCCCATCTATCAACAAAGAAGGGACCGATAGGTCATGCTCTTGGAACAAGCATG
ATAGAGTTAGGACATCTCCCAGACAAGCTTCTTGTTCAGATAATAAAATTAGGAGGGTTGAAACTCCGAAATTATATACTGTCAATTATTAAAATGGCAAGAACTATCAGTAAATTTGA
GAATAGTAAGAAACCAATTCCCAAAGTTTACAGAAAGTTGTCTATTGTTAAGGATAAAGAACTTAAGAATAGACCCATAGCAATATTTGATTATTGAAGTCAATCAGCCTTAAAACCCT
TGCATGATTCAGTAATGAATCTNNNNNNNNNNNNNNAGACCGATTTAACTTTCAATCAAGAAGGTTGTGAGGAGATACCTAGTCTTATCG

Description	Scientific Name	Max Score	Total Score	Query Cover	E value	Per. Ident	Acc. Len	Accession
Erysiphe necator mitovirus 1 isolate gpm 4 RNA-dependent RNA polymerase gene, complete cds	Erysiphe necator mitovirus 1	761	761	100%	0.0	96.41%	2571	KY420038.1
Erysiphe necator mitovirus 1 isolate PMS13_DN1896, complete genome	Erysiphe necator mitovirus 1	750	750	100%	0.0	95.96%	2707	MN599395.1

Erysiphe necator mitovirus 1 isolate 603L

Type of analysis: NCBI blastn, optimize for highly similar sequences (megablast)

CTAAGAGGGCTAGCCCCCCNNNNNNAAGTTAAAATCAAGCTGGGGTTATTATTAATATATGGGAATCGCTTTGGACATAGTAACAAGAAATCAGCTACTATAAATTATAACTTCCTAA
AATCCACTCCAGCACTTAGTGTGCTAAAATGAATCCTCAGAGAGTACAAACAAATGGCAGGTGAATCTTGCTGTTCTGTCCAGAAGGATCATGTACTTTACCTTTGGGGCTGGATTGAA
ACCAGCCTAAAACACAAAGGACTAAGTCATACAATTCTCTCACTTAAGACAATGAGGAATTGCGTGACCAAGTACATCGCAGGGGAACCAGTTAAGGTT

Description	Scientific Name	Max Score	Total Score	Query Cover	E value	Per. Ident	Acc. Len	Accession
Erysiphe necator mitovirus 1 isolate PMS13_DN1896, complete genome	Erysiphe necator mitovirus 1	599	599	100%	8e-167	98.21%	2707	MN599395.1
Erysiphe necator mitovirus 1 isolate gpm 4 RNA-dependent RNA polymerase gene, complete cds	Erysiphe necator mitovirus 1	593	593	100%	4e-165	97.92%	2571	KY420038.

Plasmopara viticola lesion associated ourmia-like virus 52 isolate 593

Type of analysis: NCBI blastn, optimize for highly similar sequences (megablast)

AGACGTTCGTGGTTCTATGCATCCTTAATCGGATTTGCTTTGAACGCGCTCTTGAACTTTCCGGTTACGCCCGTACACACCCTTGTCTTCTCAATGGTGATGACATCCTCTTTCCGGGTTG
CGATGGTCTTTATTACAGCTGGCTTCATTGTACGAAAGAAGTCGGCTTTGTAATCAATGTCAAGAAGACCATGAGATCCGCAGTTTATGGGGACTTAAATTCACAGACCTACCGTTACG
ATAAAGGTAGGTTCGTCCACAAATTCTGCTTTGGATTTCTGGGATCTGAYTCATGGAAAGAACCAGTCGGATCCCTCGCAACCCCACTTTTCGACCTCTGCGGGCAGCTTCGATTCGGA
AACGCGGCGTGGCTTYTGACCACATTCCCCGTTCGTAAGCTTCTWGCCCGCACTCCYATCCCCTTATCCTCCTTTCCCCGGCGGTGGTGGGGATTCCTTGTCAAGAAA

Description	Scientific Name	Max Score	Total Score	Query Cover	E value	Per. Ident	Acc. Len	Accession
Plasmopara viticola lesion associated ourmia-like virus 52 isolate DMG-E_27866 RNA dependent RNA polymerase gene, complete cds	Plasmopara viticola lesion associated ourmia-like virus 52	793	793	99%	0.0	97.20%	2823	MN532639.1
Erysiphe necator associated ourmia-like virus 62 isolate PMS17_156, complete genome	Erysiphe necator associated ourmia-like virus 62	566	566	99%	1e-156	88.36%	2580	MN611590.1

Plasmopara viticola lesion associated ourmia-like virus 52 isolate 605

Type of analysis: NCBI blastn, optimize for highly similar sequences (megablast)

ACGCGCTCTTGAACTTTCCGGTTACGCCCGTACACACCCCTGTCTTCTCAATGGTGATGAYATYCTTTTCCCGGGTTGCGATGGTCTTTATTACAGCTGGCTTCATTGTACGAAAGAAGT
CGGYTTTGTAATCAATGTCAAGAAGACCATGAGATCTGCAGTYTATGGGGACTTAAATTCACAGACCTACCGTTACGATAAAGGTAGGTTCGTCCACAAATTCTGCTTCGGATTTCTGG
GATCTGACTCATGGAAAGAGCCAGTCGGATCCCTCGCAACCCCACTYTTCGACCTCTGCGGGCAGCTTCGATTCGGAAACGCGGCGTGGCTTYTGACCACATTCCCCGTTCGTAAGCTT
CTTGCCCGCACTCCTATCCCCTTATCCTCCTTTCCCCGGCGGTGGTGGGGATTCCTTGTCAAGAAAAATTGGTTCCRTGGACTGGTTGATCGTGTAAAACCGACCGAG

Description	Scientific Name	Max Score	Total Score	Query Cover	E value	Per. Ident	Acc. Len	Accession
Plasmopara viticola lesion associated ourmia-like virus 52 isolate DMG-E_27866 RNA dependent RNA	Plasmopara viticola lesion associated ourmia-	785	785	100%	0.0	96.57%	2823	MN532639.1

Description	Scientific Name	Max Score	Total Score	Query Cover	E value	Per. Ident	Acc. Len	Accession
polymerase gene, complete cds	like virus 52							
Erysiphe necator associated ourmia-like virus 62 isolate PMS17_156, complete genome	Erysiphe necator associated ourmia-like virus 62	555	555	99%	3e-153	87.82%	2580	MN611590.1

Sclerotinia sclerotiorum hypovirus 1 isolate 596

Type of analysis: NCBI blastn, optimize for highly similar sequences (megablast)

TTGACGCAAACTTACCCACAACATGCCCATGGGTGGGCATTGTCTAAGGAGCGCCATAGTGATAAAGCACATCTAAAATCTTTCGATGGRCTTTATCTRGATAATAACAGTGATGAGAC
CTTAAAAGCTTATGGCGACAAAGCTTTGACTTCCAGGTCAAAGTTCAACCCCTACAACTTCATGAAACCTCGCAATATCACATGTAGATGTGGCAGACGAGGGTATGACATGAACGGT
AGATGTGAACGCTGTTTGCTAACGCAACTGGATAATGGCAGGTTGAAGATTGAGGATTTGGATTCCTTCGTGAACACTGTTTACAAAGATTTCAAACCCAAACTGAAACCTGTCTCAAA
AATGAAAACTTTCCAAAAGGAGCCTTCTGCCTTTTGGGTTCAATCACGGAAACGGTACTACTGTATAGATTCCAGATTCATCAAGAATGGTGCATATCGGCAAGTA

Description	Scientific Name	Max Score	Total Score	Query Cover	E value	Per. Ident	Acc. Len	Accession
Botrytis cinerea hypovirus 3 isolate BCI1_3, complete genome	Botrytis cinerea hypovirus 3	496	496	99%	2e-135	85.96%	10863	MN617170.1
Sclerotinia sclerotiorum hypovirus 1 strain SZ-150, complete genome	Sclerotinia sclerotiorum hypovirus 1	422	422	99%	3e-113	83.26%	10438	JF781304.1
Sclerotinia sclerotiorum hypovirus 1-A isolate SsHV1-A	Sclerotinia sclerotiorum	411	411	99%	6e-110	82.83%	10205	MF444220.1

Description	Scientific Name	Max Score	Total Score	Query Cover	E value	Per. Ident	Acc. Len	Accession
polyprotein gene, complete cds	hypovirus 1-A							
Sclerotinia sclerotiorum hypovirus 1/SX276 isolate SsHV1/SX276, complete genome	Sclerotinia sclerotiorum hypovirus 1/SX276	399	399	99%	1e-106	82.39%	10430	MT829322.1
